# Spatial transcriptional landscape of human heart failure

**DOI:** 10.1093/eurheartj/ehaf272

**Published:** 2025-05-08

**Authors:** Sang Eun Lee, Jeong Ho Joo, Hee Sang Hwang, Shang-Fu Chen, Douglas Evans, Kyoung Yul Lee, Kyung-Hee Kim, Junho Hyun, Min-Seok Kim, Sung-Ho Jung, Jae-Joong Kim, Jeong Seok Lee, Ali Torkamani

**Affiliations:** Department of Cardiology, Asan Medical Center, University of Ulsan College of Medicine, Seoul, Korea; Scripps Research Translational Institute, 3344 North Torrey Pines Court, La Jolla, CA 92037, USA; Graduate School of Medical Science and Engineering, Korea Advanced Institute of Science and Technology, Daejeon, Korea; Department of Pathology, Asan Medical Center, University of Ulsan College of Medicine, Seoul, Korea; Scripps Research Translational Institute, 3344 North Torrey Pines Court, La Jolla, CA 92037, USA; Scripps Research Translational Institute, 3344 North Torrey Pines Court, La Jolla, CA 92037, USA; Pathology Center, Seegene Medical Foundation, Seoul, Korea; Division of Cardiology, Cardiovascular Center, Incheon Sejong Hospital, Incheon, Korea; Department of Cardiology, Asan Medical Center, University of Ulsan College of Medicine, Seoul, Korea; Department of Cardiology, Asan Medical Center, University of Ulsan College of Medicine, Seoul, Korea; Department of Thoracic and Cardiovascular Surgery, Asan Medical Center, University of Ulsan College of Medicine, Seoul, Korea; Department of Cardiology, Asan Medical Center, University of Ulsan College of Medicine, Seoul, Korea; Graduate School of Medical Science and Engineering, Korea Advanced Institute of Science and Technology, Daejeon, Korea; Scripps Research Translational Institute, 3344 North Torrey Pines Court, La Jolla, CA 92037, USA; Department of Integrative Structural and Computational Biology, Scripps Research, La Jolla, CA, USA

**Keywords:** Heart failure, Cardiomyopathy, Cardiomyocytes, Endothelial cells, Spatial transcriptomics, Fibrosis, Degeneration, Myocyte disarray, Inflammation

## Abstract

**Background and Aims:**

Heart failure (HF) remains a significant clinical challenge due to its diverse aetiologies and complex pathophysiology. The molecular alterations specific to distinct cell types and histological patterns during HF progression are still poorly characterized. This study aimed to explore cell-type- and histology-specific gene expression profiles in cardiomyopathies.

**Methods:**

Ninety tissue cores from 44 participants, encompassing various forms of cardiomyopathy and control samples with diverse histological features, were analysed using the GeoMx Whole Human Transcriptome Atlas. Data on cell types, clinical information, and histological features were integrated to examine gene expression profiles in cardiomyopathy.

**Results:**

The study characterized the cellular composition of ventricular myocardium and validated the GeoMx platform’s efficiency in compartmentalizing specific cell types, demonstrating high accuracy for cardiomyocytes but limitations for endothelial cells and fibroblasts. Differentially expressed genes, including *UCHL1* from cardiomyocytes, were associated with degeneration, while *CCL14*, *ACKR1*, and *PLVAP* from endothelial cells were linked to fibrosis. Multiplex immunohistochemistry and integrative analysis of prior sc/snRNA-seq data identified a PLVAP, ACKR1, and CCL14-positive pro-inflammatory endothelial cell subtype linked to fibrosis in HF. Downregulation of ribosomal proteins in cardiomyocytes was associated with myocyte disarray in hypertrophic cardiomyopathy. Additionally, pronounced inflammatory responses were observed in end-stage HF. Combined histological and clinical analysis identified *CRIP3*, *PFKFB2*, and *TAX1BP3* as novel contributors to HF pathogenesis.

**Conclusions:**

These findings highlight the critical role of cell-enriched and histology-specific transcriptome mapping in understanding the complex pathophysiological landscape of failing hearts, offering molecular insights and potential therapeutic targets for future interventions.

## Structured Graphical Abstract

**Structured Graphical Abstract:**
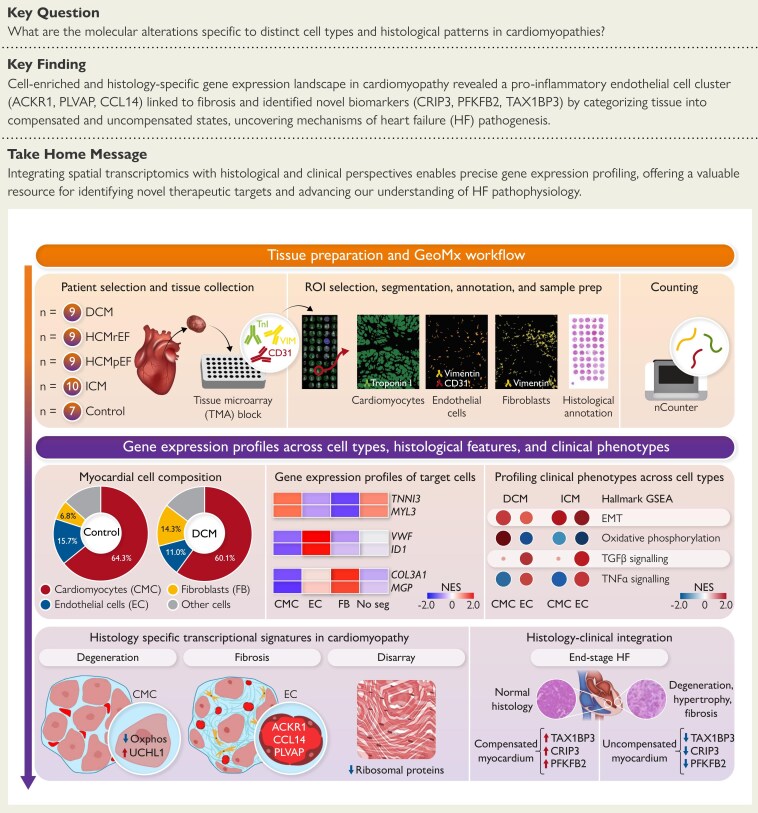

**See the editorial comment for this article ‘Unravelling heart failure cellular signalling heterogeneity with spatial transcriptomics’, by S. Tual-Chalot and K. Stellos, https://doi.org/10.1093/eurheartj/ehaf311.**

Translational perspective This study uses spatial transcriptomics to integrate gene expression profiling with histologic and clinical features in heart failure (HF). Analysis of 90 tissue cores and 178 segments from 44 study participants identified a pro-inflammatory endothelial cell cluster co-expressing *ACKR1, PLVAP*, and *CCL14*, associated with fibrosis. By categorizing tissue segments into compensated and uncompensated states, the study uncovered *CRIP3, PFKFB2*, and *TAX1BP3* as novel contributors to HF. These findings highlight the limitations of clinical phenotype-based analyses and emphasize that integrating spatial transcriptomics with histological and clinical perspectives provides a valuable resource for discovering therapeutic targets and advancing understanding of HF pathophysiology.

## Introduction

Heart failure (HF) occurs when the heart cannot pump enough blood and oxygen to sustain other organs, often due to various cardiovascular diseases.^1^ Despite recent advances in treatment, HF is still associated with high mortality and morbidity, imposing a significant global healthcare burden.^2–5^ A comprehensive understanding of the molecular mechanisms underlying the failing heart is essential for developing novel and effective therapies.^6^

While early studies using bulk RNA sequencings and microarrays provided insights into HF gene expression, they often yielded inconsistent results and lacked granularity in discerning cell-type-specific or histological expression signatures.^7–11^ Recent advances in single-cell/single-nucleus RNA sequencing (sc/snRNA-seq) have revealed the diversity of cardiac cells, with cell type-specific transcriptional programmes, and distinct disease-associated molecular alteration. These studies highlight the importance of obtaining cell-specific information and have provided a valuable resource for investigating human HF.^12–17^

However, one of the major challenges of sc/snRNA-seq is capturing the histological heterogeneity of failing heart tissue. Not only can the histology of HF vary significantly across individuals, but it can also differ within the same individual. For instance, ischaemic cardiomyopathy (ICM) ranges from normal myocardium to areas entirely replaced with fibrous tissue, depending on the time and distance from ischaemic injury and can even be diverse within the border zone.^18,19^ In hypertrophic cardiomyopathy (HCM), myocyte disarray, a pathognomonic finding related to sudden cardiac death, can occupy more than 10% of the myocardium with a preference for the hypertrophied interventricular septum, but can also be absent in some patients.^20–23^ Similarly, dilated cardiomyopathy (DCM) exhibits different degree of myocardium degeneration and fibrosis.^24,25^ While the histological features of the tissue used for sc/snRNA-seq are carefully considered, there are limitations in characterizing the histological background from where the cells originated, as the bulk tissue is lysed for the experiment.

Spatial transcriptomics has emerged to address these limitations, though most studies so far focus on normal or limited patient samples.^26,27^ To enhance our understanding of gene expression profiles within specific cardiac cell types across diverse histologic features from a spectrum of cardiomyopathies, we conducted spatial gene expression profiling in a diverse set of HF samples using the GeoMx Human Whole Transcriptome Atlas (WTA).^28^ This platform provides flexibility in selecting regions of interest (ROIs) on a given slide and is effective in compartmentalizing ROIs based on expression markers.^29–31^ By interrogating histologic phenotypes with spatial transcriptomics, we aimed to elucidate cell-type-specific molecular alterations related to histologic phenotypes, distinguish true pathologic progression in failing heart from compensatory changes, and provide a more informative resource for identifying potential biomarkers and therapeutic targets for HF.

## Methods

### GeoMx digital spatial profiling in human heart tissue

Heart tissues from 44 participants (*Table 1*) were processed under ethical approvals (Institutional Review Board (IRB) protocols at Asan Medical Center and Sejong General Hospital). Ninety tissue cores representing varied histology were arrayed on tissue microarrays (TMA), annotated by a pathologist, stained with Troponin I, Vimentin, and CD31 (PECAM-1), and analysed using the GeoMx WTA platform to generate cell-type and histology-specific gene expression profiles.

**Table 1:** Clinical summary of participants at each instance of tissue collection

	Phenotypes	Number of participants	Number of total tissue-collections^a^	Age (years)	Male (%)	BNP (pg/mL)	LVEF (%)	Chamber size (cm)
Total		44	51	55 ± 15	31 (60.8%)	1134.9 ± 1164.4	36 ± 21	57 ± 14
Clinical phenotype	DCM	9	13	49 ± 20	8 (61.5%)	1076.4 ± 1211.0	23 ± 6	68 ± 10
	ICM	10	12	62 ± 7	11 (91.7%)	1457.0 ± 1445.6	19 ± 7	62 ± 10
	HCM	18	19					
	Reduced EF (HCMrEF)	9	10	59 ± 7	5 (50.0%)	1378.4 ± 1277.1	36 ± 14	56 ± 11
	Preserved EF (HCMpEF)	9	9	50 ± 16	3 (33.3%)	688.1 ± 498.1	66 ± 5	42 ± 3
	Isolated RV failure	1	1	64	1 (100.0%)	265	65	41
	Severe lung disease	2	2	48 ± 24	1 (50.0%)	1063.5 ± 1399.4	70 ± 2	39 ± 18
	Non-cardiac death^b^	4	4	53 ± 15	2 (50.0%)			

^a^Number of total tissue-collections refers to the total count of collection events or surgical procedures from which tissue samples were obtained, including cases where multiple collections were made from the same individual across different surgeries (e.g. 7 out of 44 participants received both LVAD and heart transplants sequentially). Age, gender, BNP levels, LVEF, and chamber size are reported based on each instance of tissue collection.

^b^Autopsied after dying from non-cardiac causes. Values are presented as mean ± standard deviation (SD) and as number (%).

LVEF, left ventricular ejection fraction; DCM, dilated cardiomyopathy; ICM, ischaemic cardiomyopathy; HCM, hypertrophic cardiomyopathy; RV, right ventricle.

Data underwent quality control, batch correction, and normalization.

### Validation via cell decomposition, immunohistochemistry, and rare variant analysis

Cell-type deconvolution was performed using SpatialDecon^32^ with sc/snRNA-seq profiles.^15^ Immunohistochemistry was reviewed by blinded pathologists. Multiplex staining visualized markers to assess cell density and fibrosis relation. Rare variant analysis on UK Biobank Whole exome sequencing data explored gene associations with HF. External sc/snRNA-seq data further validated gene profiles, identifying specific endothelial subtypes.

### Quantification and statistical analysis

Differential gene expression was analysed using a linear mixed-effects model using the DREAM method^33^ to adjust for repeated sampling within individuals.

Additional methods can be found in the Supplementary material.

## Results

### Profiling of spatial transcriptomes in human cardiomyopathy

From January 2018 to April 2021, heart tissues were collected from 44 participants (mean age 55 years; 27 (61%) male). Among them, 9 had DCM, 10 had ICM, and 18 had HCM (9 with preserved EF (HCMpEF) and 9 with reduced EF (HCMrEF)) (*Table 1*; Supplementary data online, *Tables S1* and *S2*). The mean left ventricular (LV) ejection fraction (EF) was 23% for DCM, 19% for ICM, 36% for HCMrEF, and 66% for HCMpEF. Seven individuals served as controls comprising 4 individuals with non-cardiac causes of death, one case with isolated right ventricular (RV) failure, and two heart-lung co-transplantation cases for severe lung conditions (one of which also had RV failure). Although RV segments were included for quality control and initial cell-type comparisons, we excluded them from downstream analyses of failing heart and focused on LV segments. This diverse control group was intentionally selected to establish a comprehensive baseline and minimize potential biases associated with more uniform controls, such as brain-dead donor hearts,^34,35^ ensuring our findings specifically reflect disease-related molecular alterations. Demographic details and clinical characteristics are presented in *Table 1* and Supplementary data online, *Tables S1* and *S2*.

**Figure 1:**
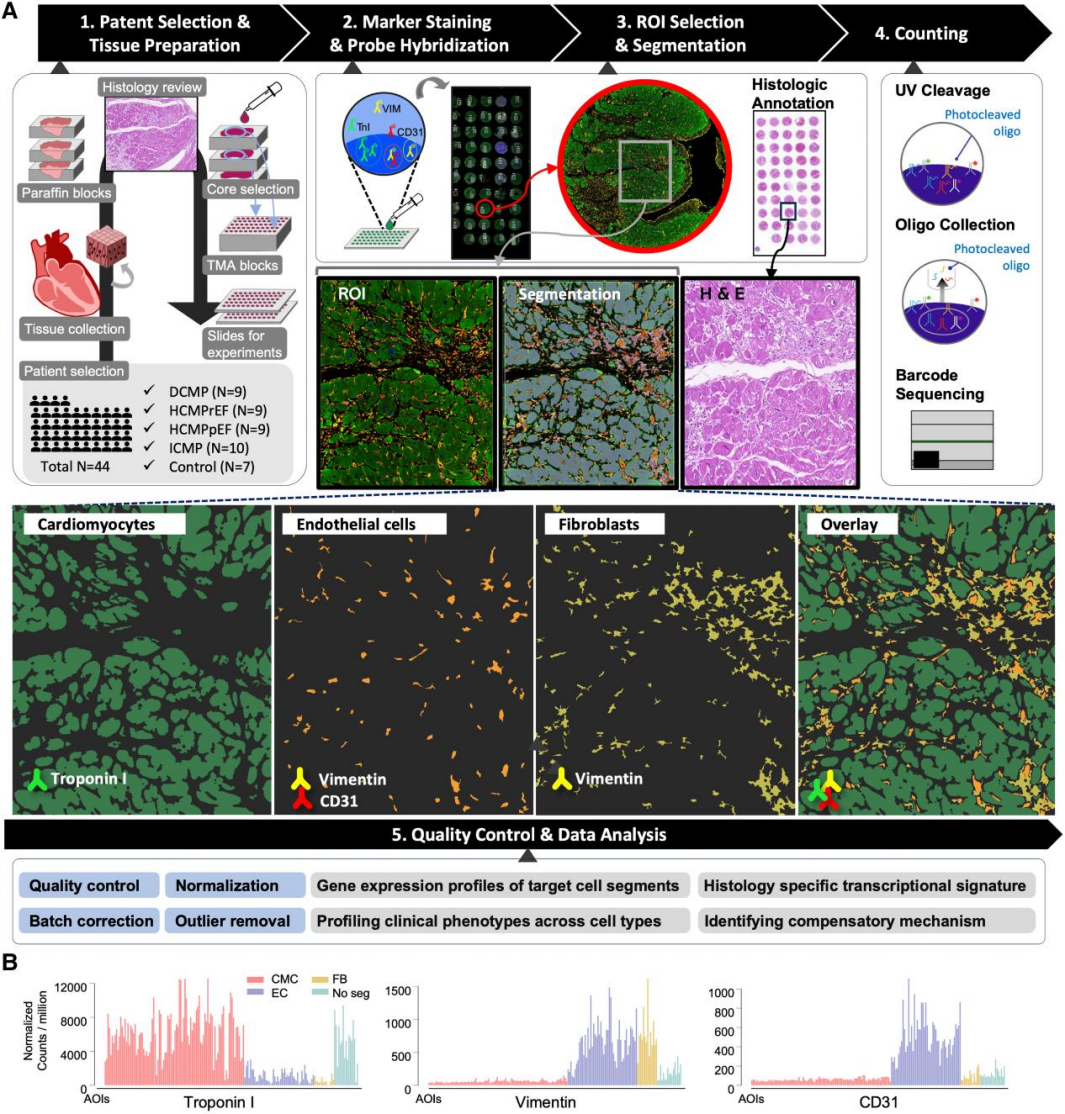
Profiling of spatial transcriptomes in human cardiomyopathy (*A*). Schematic Figures depicting the experimental process: 1. Patient selection and tissue preparation: Selection of patients, tissue harvest, selecting cores based on histology, preparation of tissue microarray (TMA) blocks, and slicing the TMA blocks for experiments. 2. Marker staining and probe hybridization: Staining of markers for target cells (antibodies against Troponin I (TnI), CD31, and Vimentin (VIM)) and probe hybridization. 3. Region of interest (ROI) selection and segmentation: An example of selecting ROIs based on paired H&E staining, and segmentation of areas of interest (AOIs) of target cells based on immunofluorescence staining. The boxes indicate the ROIs. 4. Counting: Execution of the GeoMx Digital Spatial Profiling experiment to yield count data. 5. Quality Control and Data Analysis: From quality control to downstream analysis. (*B*) Bar graphs showing the normalized counts per million of transcripts for each gene in AOIs classified by cardiomyocytes (CMC), endothelial cells (EC), fibroblasts (FB), and non-segmented ROIs (No_Seg). Total sample number is 178. Detailed number of AOIs per cell-type or non-segmented ROI is described in *Table 2*. DCM, dilated cardiomyopathy; ICM, ischaemic cardiomyopathy; HCMpEF, hypertrophic cardiomyopathy with preserved ejection fraction; HCMrEF, hypertrophic cardiomyopathy with reduced ejection fraction)

*Figure 1A* outlines the selection and profiling process for spatial transcriptomes in human cardiomyopathy. A pathologist reviewed hematoxylin and eosin (H&E)-stained slides from multiple heart regions, selecting 90 cores spanning a range of histology and diagnoses. These cores were placed on two TMA blocks, and the slides from the blocks were incubated with ribonucleic acid (RNA) probes and stained with fluorescent-labelled Troponin I, Vimentin, and CD31 antibodies. In total, 178 segments were identified by the pathologist targeting specific cell types: 98 cardiomyocyte, 49 endothelial cell, and 14 fibroblast segments, alongside 17 non-segmented regions of interest (ROIs) based on both H&E and immunofluorescent staining characteristics (*Table 2*). Cardiomyocyte segments were Troponin I-positive, endothelial segments were Vimentin and CD31 double-positive, but Troponin I-negative, and fibroblast segments were Vimentin-positive, CD31 and Troponin I double-negative. This antibody-based segmentation strategy was validated through multiple approaches. First, we confirmed the marker gene expression patterns using sc/snRNA-seq data,^15,36^ demonstrated that Vimentin is substantially expressed in endothelial cells (see Supplementary data online, *Figure S1A*–*S1D*). Second, we emulated our segmentation strategy using the snRNA-seq data by performing pseudo-bulk principal component analysis (PCA) at the patient level, aggregating single nuclei based on marker gene expression. This approach aimed to replicate our antibody-based cell selection strategy. The results demonstrated clear segregation of cell types, whether they were Vimentin and CD31 double-positive (see Supplementary data online, *Figure S1E*) or CD31 single-positive (see Supplementary data online, *Figure S1F*). Third, our antibody staining results for GeoMx experiment and Vimentin co-localization with CD31 were reviewed by a pathologist (see Supplementary data online, *Figure S2A–S2F*) and validated by additional multiplex immunohistochemistry and automated cell countering showing 91% of CD31 positive cells were Vimentin = positive (see Supplementary data online, *Figure S2G–S2P*). Lastly, we analysed the biologic relevance of our target cell segmentation exploring differentially expressed genes across cell types.

**Table 2:** Summary of segments (area of interest, AOI)

		Total	LV no segmentation	LV cardiomyocytes	LV endothelial cells	LV fibroblasts	Right ventricle
Number of ROIs		117					
Number of AOIs		178	15	81	44	11	27
Segment type	Full ROI	17	15	0	0	0	2
	Cardiomyocytes	98	0	81	0	0	17
	Endothelial cells	49	0	0	44	0	5
	Fibroblasts	14	0	0	0	11	3
Clinical phenotype 1	Non-end-stage	66	4	34	17	2	9
	End-stage	112	11	47	27	9	18
Clinical phenotype 2	Control	11	1	6	3	0	1
	DCM	40	5	15	10	4	6
	ICM	34	2	17	7	4	4
	HCM	72	6	37	21	3	5
	With preserved EF	31	1	18	10	2	0
	With reduced EF	41	5	19	11	1	5
	Severe lung disease^a^	8	0	2	0	0	6
	Isolated RV failure	13	1	4	3	0	5
After LVAD	Yes	28	5	10	4	4	5
Histologic phenotype	Normal	48	4	23	11	0	10
	Diseased	130	11	58	33	11	17
Degeneration	None	51	4	26	11	0	10
	Mild	78	5	33	18	5	17
	Moderate	35	3	18	11	3	0
	Severe	14	3	4	4	3	0
Hypertrophy	None	115	10	53	25	5	22
	Mild	48	4	21	14	4	5
	Moderate	8	0	4	3	1	0
	Severe	7	1	3	2	1	0
Fibrosis	None	99	9	49	24	0	17
	Mild	53	4	23	12	6	8
	Moderate	18	1	7	6	2	2
	Severe	8	1	2	2	3	0
Disarray	None	132	12	62	33	9	16
	Mild	33	2	15	7	1	8
	Severe	13	1	4	4	1	3
Peri-infarction area		15	1	8	2	2	2
Fixation interval^b^	Short	97	7	53	30	4	3
	Long	81	8	28	14	7	24
ROI size	Large	170	15	75	43	11	26
	Small	8	0	6	1	0	1

^a^Requiring lung and heart transplantation.

^b^Short fixation interval: <4 h, long fixation interval: 4–12 h.

LV, left ventricle; ROI, region of interest; AOI, area of interest; DCM, dilated cardiomyopathy; ICM, ischaemic cardiomyopathy; HCM, hypertrophic cardiomyopathy; EF, ejection fraction; RV, right ventricle; LVAD, left ventricular assistant device.

Following data acquisition, 12 800 out of 18 677 genes met quality control standards and were included in further analysis. Batch effects across tissue slides were corrected with *ComBat-seq* (see Supplementary data online, *Figure S3*)^37^ and counts were normalized using the third quartile (Q3) method. RNA expressions of each marker in the corresponding segments and non-segmented ROIs showed clear separation (*Figure 1B*).

Our analysis aimed to validate data integrity and reveal cell type- and histology-specific gene expression patterns by: (i) evaluating cell segmentation and gene expression in each compartment, (ii) comparing cardiomyocyte and endothelial transcriptional profiles across clinical diagnoses, with emphasis on HCMpEF vs HCMrEF to investigate mechanisms of HCM progression, (iii) examining histology-specific transcriptional differences in degenerated, hypertrophic, fibrotic, and disarrayed regions, and (iv) identifying molecular profiles associated with compensatory states by categorizing segments based on histology and clinical features.

### Efficacy of segmentation and gene expression profiles in cardiac cell types

**Figure 2:**
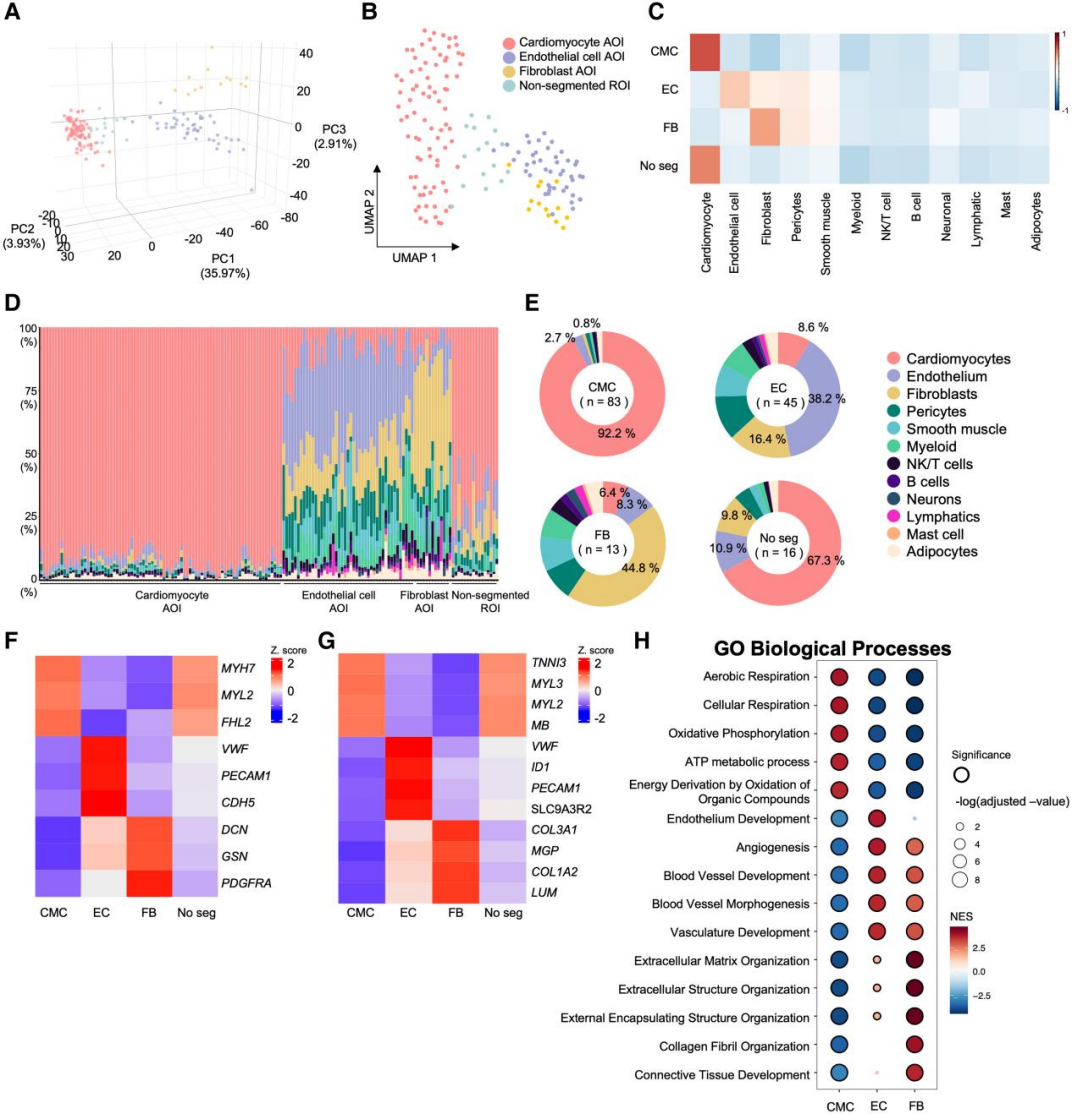
Overview of gene expression profiles according to cell types (*A*). Three-dimensional principal component analysis (PCA) plot showing the distribution of segments across the first three principal components (PC1, PC2, and PC3), annotated by cell types. (*B*) Uniform manifold approximation and projection (UMAP) representation of each segment (area of interest, AOI) and non-segmented ROI, coloured by cell types. (*C*) Heatmap illustrating the correlation between log count per million from our data and averaged expression values from single-nucleus RNA sequencing (snRNA-seq) data of heart tissue by Koenig *et al*., for differentially expressed genes (DEGs) identified in the snRNA-seq analysis. (*D*) Bar plot showing the proportion of cell types in each segment, calculated by spatial deconvolution (*E*). Donut plot depicting the average proportion of cell types in different types of segments, calculated by spatial deconvolution. (*F, G*). Heatmap demonstrating expressions of curated marker genes from the literature (*F*) and the top four genes with the highest log fold change (logFC) from DEGs comparing each segment to the others in each segment and non-segmented ROI (*G*). The colour represents the z-score. *(H*) Dot plots representing the normalized enrichment score (NES) of gene ontology biological processes (GOBP) for each cell type. Dot size indicates the -log10 (adjusted *P*-value, Benjamini–Hochberg FDR), and the colour reflects the NES score from gene set enrichment analysis (GSEA). The total number of samples is 157, with CMC: 83, EC: 45, FB: 13, and No_Seg: 16, excluding 8 small ROIs and 13 outliers on the PCA plot. CMC, cardiomyocytes; EC, endothelial cells; FB, fibroblasts; No_Seg, non-segmented ROIs. The number of samples from Koenig’s data is 38, and the total number of nuclei included in this analysis is 203 333

PCA and uniform manifold approximation and projection (UMAP) effectively visualize the distinct gene expression profiles among cardiomyocytes, endothelial cells, fibroblasts, and non-segmented regions (*Figure 2A* and *B*). Non-segmented regions lay between cardiomyocytes and other cell types, with endothelial cells and fibroblasts closely positioned. Notably, UMAP reveals that endothelial cells from cardiomyopathy patients scatter more and are nearer to fibroblasts, whereas those from control tissues are more distinctly separated from fibroblasts (see Supplementary data online, *Figure S4*). This pattern reflects endothelial-mesenchymal transition in cardiac fibrosis, a phenomenon supported by existing studies.^38^ These gene expression profiles across the three cell types exhibit strong concordance with previous snRNA-seq data (*Figure 2C*). PCA linked cell type to PC1 and PC4, fibrosis grade to PC1, and clinical phenotype to PC2 and PC5, with no significant batch effects from fixation interval and TMA block (see Supplementary data online, *Figure S5A* and *S5B* and *Table S3*). SpatialDecon analysis,^32^ utilizing previously published gene profiles for each cardiac cell type,^15^ demonstrated effective segmentation across cell types (*Figure 2D* and *E*). Cell composition in non-segmented regions differed from published sc/snRNA-seq data, showing 67% cardiomyocytes, 10% fibroblasts, and 11% endothelial cells, compared with 17%–49%, 16%–47%, and 8%–23%, respectively, in prior sc/snRNA-seq studies.^15,16^ Specifically, control tissue contained only 6.8% fibroblasts, which increased with degeneration and fibrosis (see Supplementary data online, *Figure S6*). Immunofluorescence staining further supported this, with Vimentin primarily co-localizing with CD31, indicating an endothelial predominance (see Supplementary data online, *Figure S2A–S2C*). The discrepancy may stem from the use of transmural tissue in sc/snRNA-seq studies, which include fibroblast-rich epicardium and vessel-dense areas, along with variations in cell dissociation efficiency. In contrast, our approach specifically targeted distinct histological regions within the ventricular myocardium, allowing us to capture a more precise and representative cell composition in this area. However, the low fibroblast proportion limited fibroblast segment analysis on the GeoMx platform, leading us to focus on cardiomyocyte and endothelial segments. Cardiomyocyte segmentation was effective, enriching cardiomyocytes from 67% to 92% and minimizing other cell types (*Figure 2D* and *E*). However, achieving pure compartmentalization for endothelial cells and fibroblasts proved more challenging. Endothelial cell enrichment rose from 11% to 38%, though some vessel-associated cells remained. Similarly, Vimentin-positive segmentation increased fibroblast enrichment from 10% to 45%, with some non-fibroblast, vessel-associated cells remaining.

We analysed differentially expressed genes across cell types (see Supplementary data online, *Figure S7* and *Table S4*, https://cardiogene.shinyapps.io/spatial_cmp/). Curated marker genes from the literature were specifically expressed in target segments (*Figure 2F*).^16^ Notably, genes that were significantly overexpressed in each segment also overlapped substantially with the curated marker genes, demonstrating cell-type-specific expression patterns (*Figure 2G*). Cardiomyocytes showed high expression of genes related to muscle contraction (e.g. *TNNI3*, *MYL2*, and *MYL3*) and energy metabolism (e.g. *MB*), highlighting their contractile and metabolic roles.^39^ Endothelial cells expressed genes such as *VWF and PECAM1*, involved in coagulation and vascular function, along with *ID1 and SLC9A3R2*, which support vascular remodelling and homeostasis.^40^ Fibroblasts displayed genes related to extracellular matrix and fibrosis (e.g. *COL3A1*, *COL1A2*, *LUM*, and *MGP)*.^41–44^ Gene ontology (GO) enrichment aligned with these findings, showing positive enrichment of pathways in energy metabolism and respiration for cardiomyocytes, vascular development and angiogenesis for endothelial cells, and extracellular matrix organization for fibroblasts (*Figure 2H*). These results validate our segmentation approach in accurately capturing cell-type-specific gene expression and biological processes.

### Cell type-dependent regulation of gene expression in cardiomyopathies

**Figure 3:**
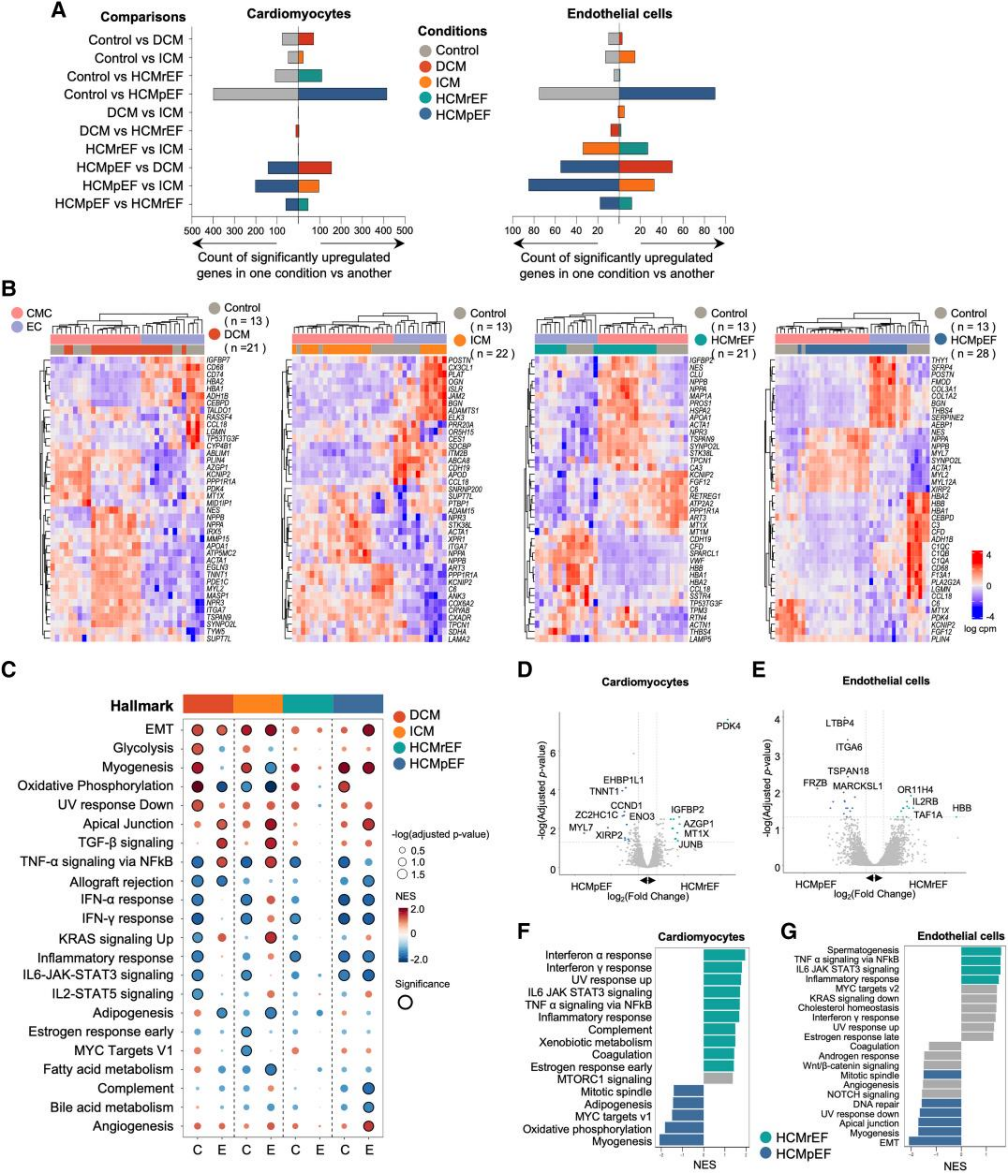
Cell type-dependent regulation of gene expression profiles in cardiomyopathies (*A*). Bar plots depicting the number of differentially expressed genes (DEGs) for each pairwise comparison among cardiomyopathies and control, categorized by cell type (cardiomyocytes on the left, endothelial cells on the right). For each comparison, bars on the left indicate genes significantly up-regulated in the first group, and bars on the right indicate genes significantly up-regulated in the second group. The colour of each bar corresponds to a specific clinical phenotype (see legend), and the height represents the total count of significantly up-regulated genes. (*B*) Heatmap showing the top and bottom 20 DEGs between each cardiomyopathy and control, clustered by cell type and disease presence (the sample numbers are in parentheses). (*C*) Dot plots depicting Hallmark gene set enrichment for differential expression between each cardiomyopathy vs control in cardiomyocytes (*C*) and endothelial cells (*E*). The size of the dots indicates the -log10 (adjusted *P*-value, Benjamini–Hochberg FDR), and the colour reflects the NES score from gene set enrichment analysis (GSEA). (*D-E*). Volcano plot displaying the log fold change (logFC) and two-sided *P*-value from the differential expression analysis between HCMrEF and HCMpEF in cardiomyocyte segments (*D*) and in endothelial cell segments (*E*). (*F, G*). Bar plot depicting Hallmark gene set enrichment for differential expression between HCMrEF and HCMpEF in cardiomyocyte segments (*F*) and endothelial cell segments (*G*). The x-axis indicates the NES from GSEA. Green and blue bars represent gene sets activated in HCMrEF and HCMpEF, respectively, with a Benjamini–Hochberg FDR *P*-value < .05. Pathways with a Benjamini–Hochberg FDR > 0.05 are coloured in grey. DCM, dilated cardiomyopathy; ICM, ischaemic cardiomyopathy; HCMpEF, hypertrophic cardiomyopathy with preserved ejection fraction; HCMrEF, hypertrophic cardiomyopathy with reduced ejection fraction. The number of samples used in the DEG analysis for cardiomyocytes are as follows: control (7), DCM (13–14), ICM (16), HCMrEF (11–13), and HCMpEF (18). For endothelial cells, the numbers are as follows: control (6), DCM (8–10), ICM (7), HCMrEF (6–8), and HCMpEF (10). The number of samples varies in the same group due to the outlier selection process for each comparison, and the detailed number of samples used in each comparison is described in Supplementary data online, *Table S19*)

By comparing HF and control samples, we identified genes differentially expressed in cardiomyocytes and endothelial cells per clinical diagnosis (*Figure 3A*;  Supplementary data online, *Figure S8*, Supplementary data online, *Tables S5* and *S8*, https://cardiogene.shinyapps.io/spatial_cmp/). HCMpEF showed the most distinct expression pattern compared with controls and other cardiomyopathies, regardless of cell type. In contrast, comparisons among reduced ejection fraction cardiomyopathy subtypes (DCM, ICM, and HCMrEF) exhibited minimal differential expression, consistent with previous sc/snRNA-seq and proteomic analyses suggesting a convergence towards a common transcriptional profile in advanced cardiomyopathy.^14,45^ A heatmap of differentially expressed genes between each cardiomyopathy and controls were primarily clustered based on cell type, and then by disease, indicating that cardiomyopathy-related gene expression is differentially regulated across cell types (*Figure 3B*). Notably, with similar sample sizes, the heatmap for DCM (a primarily cardiomyocyte disease) shows more distinctive gene expression patterns in cardiomyocytes compared with endothelial cells. Conversely, in ICM (a primarily vessel-origin disease), endothelial cells display a more distinct gene expression pattern than cardiomyocytes. Specifically, ICM endothelial cells show up-regulation of genes like *POSTN*, *CX3CL1*, and *BGN*, which are associated with apoptosis, cardiac function, and adaptive remodelling post-myocardial infarction.^46–48^ This unique ICM endothelial signature, distinct from gene expression profiles in other end-stage HF and in ICM cardiomyocytes, suggests endothelial-derived factors as potential biomarkers or therapeutic targets.

We applied gene-set enrichment analysis (GSEA) to cardiomyocyte and endothelial cell gene expression data from each cardiomyopathy vs controls to assess cell type-specific pathway regulation. Our analysis revealed both common and divergent gene-set regulation across cell types (*Figure 3C*). For instance, the epithelial-mesenchymal transition gene set showed consistent up-regulation in both cardiomyocytes and endothelial cells across most cardiomyopathies. However, oxidative phosphorylation showed opposite patterns, with up-regulation in cardiomyocytes and downregulation in endothelial cells, particularly in DCM. Similarly, TNFα signalling via NFκB displayed differential expression between cell types, being down-regulated in cardiomyocytes and up-regulated in endothelial cells. This reciprocal regulation between cardiomyocytes and endothelial cells was even more pronounced in ICM for inflammation-related gene sets. Notably, this inverted expression pattern between cardiomyocytes and endothelial cells was less pronounced in HCMpEF or HCMrEF.

An intriguing aspect of our clinical phenotype comparison lies in the differences between HCMpEF and HCMrEF. In HCM, progressive systolic HF can lead to HCMrEF—also known as end-stage or burned-out HCM—a severe, treatment-refractory state often necessitating heart transplantation,^49,50^ underscoring the need to understand its molecular basis. Our analysis identified unique gene regulation in cardiomyocytes and endothelial cells (*Figure 3D* and *E*;  Supplementary data online, *Tables S5* and *S6*, https://cardiogene.shinyapps.io/spatial_cmp/). Notably, *IGFBP2* was significantly up-regulated in HCMrEF relative to HCMpEF, as confirmed by immunohistochemistry (see Supplementary data online, *Figure S9*). As an IGF1 inhibitor involved in inflammation and fibrosis,^51^ IGFBP2 has been proposed as a diagnostic and prognostic marker for HF.^52,53^ In our analysis, while natriuretic peptides showed no significant differences between the groups, *IGFBP2* exhibited substantial variation, indicating its potential as a novel marker or therapeutic target for fibrosis- and inflammation-driven progression. GSEA further revealed marked up-regulation of inflammation-related gene sets in both cardiomyocytes and endothelial cells of HCMrEF (*Figure 3F* and *G*), whereas oxidative phosphorylation and myogenesis—up-regulated in HCMpEF vs controls (*Figure 3C*)—were significantly down-regulated in HCMrEF (*Figure 3F*). These findings suggest that progression to end-stage HCM involves diminished compensatory myogenesis and oxidative phosphorylation alongside enhanced inflammation.

### Spatial gene expression landscape differences by histology

**Figure 4:**
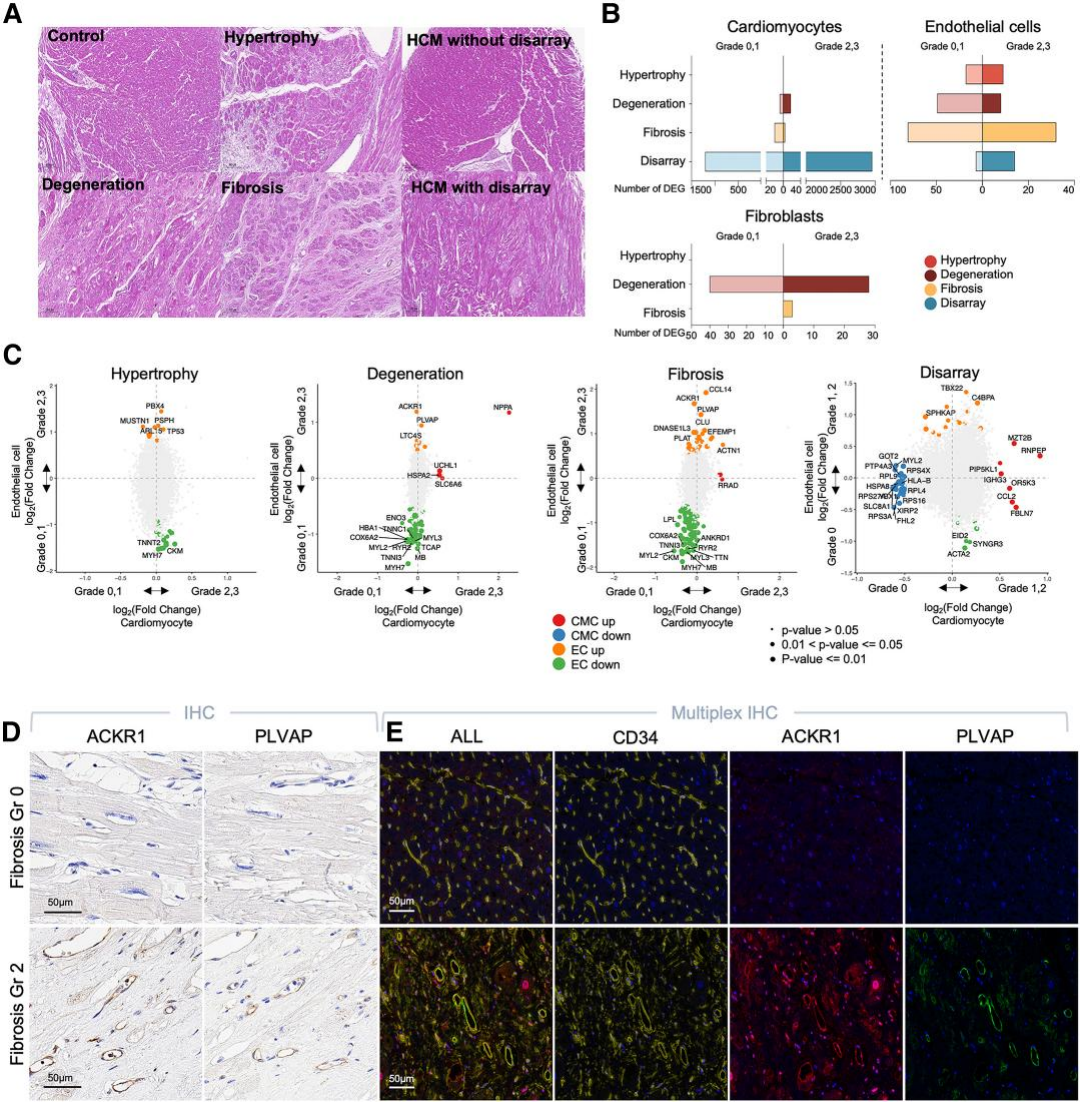

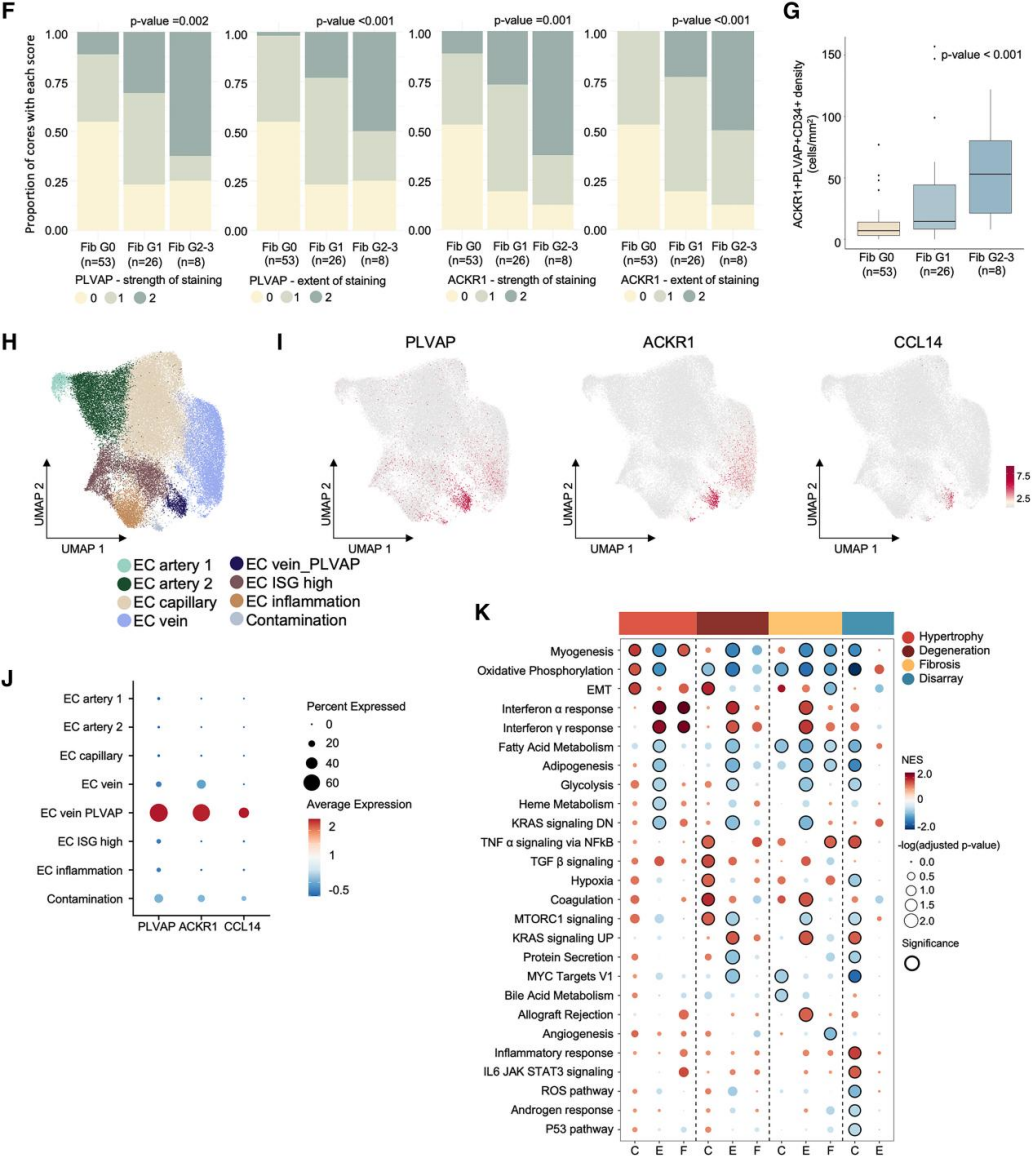
Spatial gene expression landscape differences by histology (*A*). Examples of H&E staining showing typical histological features in cardiomyopathy, including control, hypertrophy, degeneration, fibrosis, HCM without disarray, and HCM with disarray. (*B*) Bar plot depicting the number of differentially expressed genes (DEGs) from the comparison between grades 0 and 1 vs grades 2 and 3 of each histology (grade 0 vs 1–2 in disarray) in cardiomyocyte, endothelial, and fibroblast segments. The height of each bar represents the number of positive DEGs for each condition. (*C*) Scatter plots displaying the log fold change (logFC) in either cardiomyocyte segmentation (x-axis) or endothelial segmentation (y-axis) from the DEGs between grade 0 and 1 vs grade 2 and 3 of each histology (grade 0 vs 1–2 in disarray). The size of dots indicates the two-sided *P*-value, categorized into three groups based on thresholds of 0.05 and 0.01. Dot colours represent the direction of changes: red indicates genes up-regulated in CMC, blue indicates genes down-regulated in CMC, orange indicates genes up-regulated in EC, and green indicates genes down-regulated in EC. (*D*) Examples of immunohistochemistry staining of tissue microarray (TMA) blocks with ACKR1, and PLVAP. (*E*) Examples of multiplex immunohistochemistry staining of TMA blocks with CD34, ACKR1, PLVAP, and nuclei marker DAPI. (*F*) Bar graphs of PLVAP and ACKR1 staining scores (staining strength and extent respectively) according to fibrosis grades. *P*-values are from χ^2^ test and total number of cores included in analysis is 87. The sample numbers are indicated in parentheses. (*G*) Boxplot representing density of cells co-expressing CD34, ACKR1, and PLVAP, stratified by fibrosis grade. Statistical analyses were performed using a one-way ANOVA test. The sample numbers are indicated in parentheses. (*H*) Uniform manifold approximation and projection (UMAP) representation of sub-clustered endothelial cells from single-cell/single-nucleus RNA sequencing data of heart tissue by Koenig *et al*. (*I*) UMAP plots showing the normalized expression of fibrosis-associated genes (PLVAP, ACKR1, and CCL14) in endothelial cells. (*J*) Dot plot demonstrating the specific expression of PLVAP, ACKR1, and CCL14 in the EC vein PLVAP subcluster. (*K*) Dot plots depicting Hallmark gene set enrichment for differential expression between grade 0 and 1 vs grade 2 and 3 of each histology (grade 0 vs 1–2 in disarray) in cardiomyocytes (*C*), endothelial cells (*E*) and fibroblast (*F*) segments. The size of the dots indicates the -log10 (adjusted *P*-value, Benjamini–Hochberg FDR), and the colour reflects the normalized enrichment score (NES) from gene set enrichment analysis (GSEA). H&E, Hematoxylin and Eosin; ROI, regions of interest; CMC, cardiomyocytes; EC, endothelial cells; PLVAP, plasmalemma vesicle-associated protein; ACKR1, atypical chemokine receptor 1; ANOVA, analysis of variance. The number of samples used in the comparisons of DEGs for cardiomyocytes is: hypertrophy (62 vs 6), degeneration (40 vs 20), fibrosis (60 vs 8), and disarray (8 vs 9). For endothelial cells: hypertrophy (35 vs 4), degeneration (27 vs 12), fibrosis (32 vs 7), and disarray (5 vs 4). For fibroblasts: hypertrophy (9 vs 2), degeneration (5 vs 6), and fibrosis (6 vs 5). Detailed numbers of samples used in each comparison are described in Supplementary data online, *Table S19*. The number of samples from Koenig’s data is 45 and the total number of cells/nuclei included is 49 382

HF exhibits substantial histological variation both between and within individuals. Spatial transcriptomics enables correlation of gene expression profiles with specific histologic features. We analysed genes differentially expressed in association with key HF histologic features: hypertrophy, degeneration, and fibrosis (*Figure 4A–C*;  Supplementary data online, *Figure S10* and *Tables S9*–*S11*, https://cardiogene.shinyapps.io/spatial_cmp/). Degeneration had the highest number of differentially expressed genes in cardiomyocytes and fibroblasts, while fibrosis showed the most in endothelial cells. Hypertrophy exhibited the fewest differentially expressed genes across all cell types (*Figure 4B*). The correlation between gene expression variation explained by histological features and clinical diagnosis was low (see Supplementary data online, *Figure S11*). Key genes linked to these histologic features are highlighted in *Figure 4C*. For instance, *UCHL1*, significantly up-regulated in cardiomyocytes associated with degeneration, was further validated through immunohistochemistry (see Supplementary data online, *Figure S9*). This gene has been linked to HF development in animal models,^54,55^ though in humans it has only been reported for neurodegenerative disorders.^56^

Among the genes differentially expressed in endothelial cells related to fibrosis, *ACKR1,* encoding DARC expressed on venular endothelial cells, facilitate leucocyte migration through chemokine transcytosis.^57^ Its mutation in humans has been associated with the progression of liver fibrosis and cirrhosis.^58^ Plasmalemma vesicle-associated protein (*PLVAP*) is an endothelial cell-specific protein that forms the stomatal and fenestral diaphragms of blood vessels and regulates basal permeability, leucocyte migration, and angiogenesis.^59^ Our immunohistochemistry staining of TMA slides for ACKR1 and PLVAP confirmed significant correlations between fibrosis grade and both the strength and extent of ACKR1 or PLVAP expression in endothelial cells (*Figure 4D* and *F*, Supplementary data online, *Figure S12A*). ACKR1 and PLVAP expressions were also significantly correlated with each other (Spearman’s correlation, *P*-values <.001 for both staining strength and extent; rho 0.68 and 0.64, respectively). Multiplex immunohistochemistry confirmed their co-localization in endothelial cells from fibrotic tissue, with cell density significantly correlating with fibrosis grade (*Figure 4E and G*) and LVEF <30%, but not with DCM diagnosis (see Supplementary data online, *Figure S12B* and *S12C*). This aligns with recent studies in liver cirrhosis, where ACKR1 and PLVAP double-positive endothelial cells expanded within the fibrotic niche, enhancing leucocyte transmigration.^60^ Additionally, *CCL14*, a marker of coronary artery endothelial cells with *ACKR1*,^61^ was significantly overexpressed in endothelial cells of fibrotic regions in our spatial transcriptomics (*Figure 4C*) and immunohistochemistry (see Supplementary data online, *Figure S9*). To validate the presence of ACKR1 and PLVAP double-positive, CCL14-expressing endothelial cells, we analysed sc/snRNA-seq data from DCM and controls.^15^ Eight endothelial cell sub-clusters were identified (*Figure 4H*), with the ‘Endothelial cell vein PLVAP’ cluster co-expressing PLVAP, ACKR1, and CCL14 (*Figure 4I* and *J*). This cluster tended to increase in DCM without statistical significance (see Supplementary data online, *Figure S13A*), mirroring our multiplex immunohistochemistry results (see Supplementary data online, *Figure S12B*). Genes highly expressed in this cluster were enriched for inflammatory gene sets such as IFNγ response and TNFα signalling via NF-kB (see Supplementary data online, *Figure S13B*). Complementing the Hallmark gene-set analysis (*Figure 4K*), which showed up-regulation of inflammation-related gene sets in endothelial cells across all histologic features, these findings support the role of vascular inflammation and endothelial activation in HF progression.^62^ Furthermore, significant ligand-receptor interactions involving *ACKR1* or *CCL14* on the endothelial side correlated with fibrosis severity (see Supplementary data online, *Figure S14*).

Finally, our histologic analysis highlights a molecular signature linked to myocyte disarray in HCM. Myocyte disarray, a pathognomonic feature associated with sudden cardiac death in HCM, is often confined to specific myocardial regions, making it difficult to isolate tissue with this feature for bulk or sc/snRNA-seq studies. However, using GeoMx, we identified regions with disarray in HCMpEF and explored their gene expression profiles, revealing significant downregulation of ribosomal proteins in disarrayed cardiomyocytes (*Figure 4C*). Although myocyte disarray has been understudied, and few molecular pathways are defined, prior studies support the relevance of our findings. One study linked mutation in ribosomal protein to HCM in humans^63^ and another reported that haploinsufficiency in ribosomal protein in Drosophila could result in HF.^64^ While preliminary, our results may lay the groundwork for future research into the pathogenesis of myocyte disarray in HCM and open new research avenues.

### Unveiling transcriptional landscape of cardiomyopathy progression integrating clinical and histologic features

**Figure 5:**
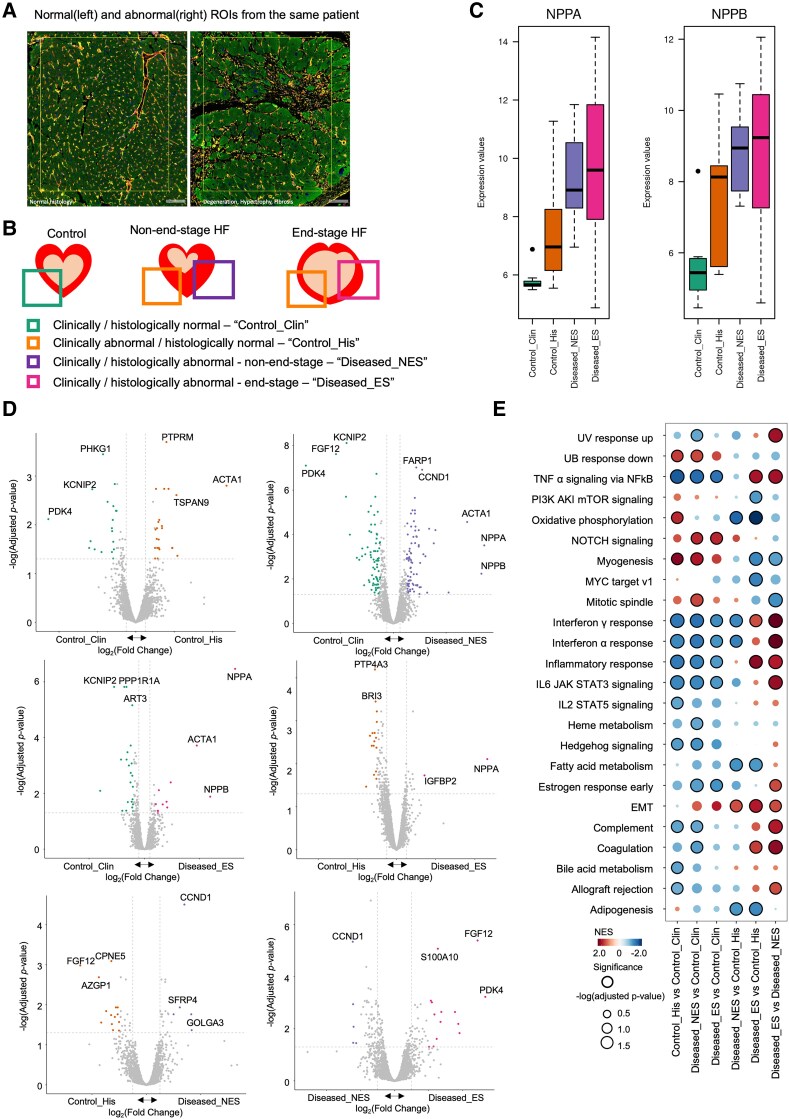
Transcriptional landscape of cardiomyopathy progression integrating clinical and histologic features (*A*). An example of the region of interest with normal (left) and abnormal histology (right) from the same patient. (*B*) Schematic Figure illustrating four groups for comparison: tissues with normal histology from control group (Control_Clin, green), tissues with normal histology from cardiomyopathy patients (Control_His, orange), tissues with abnormal histology from non-end-stage HF patients (Diseased_NES, purple), and end-stage HF (Diseased_ES, pink). (*C*) Bar graphs depicting the expression levels of NPPA and NPPB in each group. (*D*) Volcano plots displaying the log fold change (logFC) (x-axis) and two-sided *P*-value (y-axis) from the differential expression analysis in the comparison across different groups of cardiomyocyte segmentation. The colour of the dots corresponds to the groups defined in (*B*), indicating genes that are significantly increased in each group. (*E*) Dot plots depicting the Hallmark gene set for differential expression across the groups of cardiomyocyte segmentation. The size of the dots indicates the -log10 (adjusted *P*-value, Benjamini–Hochberg FDR), and the colour reflects the NES score from gene set enrichment analysis (GSEA). (The number of samples in each group for cardiomyocytes is as follows: Control_Clin (7), Control_His (9), Diseased_ES (38), and Diseased_NES (15))

Cardiac tissue from cardiomyopathy can exhibit normal histology during under-compensation or before irreversible structural changes (*Figure 5A*). We categorized cardiomyopathy tissue segments into two histologic groups—normal and abnormal—and grouped advanced cardiomyopathies (DCM, ICM, and HCMrEF) together relative to HCMpEF, based on both our analysis (*Figure 3A*) and previous literature showing convergent gene expression in end-stage HF, regardless of initial aetiology.^14,45^ This approach resulted in four distinct groups integrating clinical and histologic features: normal histology from controls (Control_Clin), normal histology from cardiomyopathy (Control_His), abnormal histology from non-end-stage HF (Diseased_NES), and abnormal histology in end-stage HF (Diseased_ES) (*Figure 5B*). The gradual increase in *NPPA* and *NPPB* expression from Control_Clin to Diseased_ES supports the relevance of this grouping in representing HF progression (*Figure 5C*). By comparing these groups, we identified molecular profiles associated with different states of HF progression in both cardiomyocytes (*Figure 5D*;  Supplementary data online, *Tables S12* and *S13*) and endothelial cells (see Supplementary data online, *Figure S15A* and *Tables S14* and *S15*, https://cardiogene.shinyapps.io/spatial_cmp/).

We hypothesized that genes reciprocally regulated between compensated (Control_His) and uncompensated (Diseased_ES) states might be associated with HF development and progression. In cardiomyocytes, 12 genes up-regulated in Control_His compared with Control_Clin became down-regulated in Diseased_ES relative to Control_His. To validate their association with HF, we conducted a rare-variant analysis of the UK biobank cohort on these genes, revealing *TAX1BP3, CRIP3, and PFKFB2* as linked to HF development (see Supplementary data online, *Table S16* and *Figure S16*). Notably, mutation in *TAX1BP3* has been associated with DCM in a family,^65^ and *PFKFB2* encoding Phosphofructokinase 2, a primary regulator of cardiac glycolysis, has been reported to mitigate hypoxia-related myocardial injury.^66^ These results suggest that molecular alterations between each state of HF could reveal the underlying process involved in the progression from compensated to uncompensated status leading to HF.

Hallmark gene-set analysis yielded complementary insights into HF development and progression. Myogenesis and oxidative phosphorylation were up-regulated in Control_His compared with Control_Clin but decreased in Diseased_ES compared with Control_His and Diseased_NES (*Figure 5E*). These gene sets may represent compensating mechanisms to maintain normal morphological features or at least preserve EF, which fail during progression to uncompensated end-stage. In contrast, inflammation-related gene sets were down-regulated in Control_His compared with Control_Clin, but significantly up-regulated in Diseased_ES compared with Control_His and Diseased_NES. These findings suggest that exacerbation of inflammation and failure of myogenesis may represent a tipping point of irreversible structural changes leading to end-stage HF.

This categorization also allowed us to evaluate gene expression differences related to treatment and comorbidities in groups where their prevalence is balanced (see Supplementary data online, *Tables S13* and *S15*), which would otherwise be challenging due to clinical phenotype variability (see Supplementary data online, *Tables S7* and *S8*). Our analysis revealed that in tissues with abnormal histology from end-stage HF (Diseased_ES), gene expression differences attributed to treatments or comorbidities were significantly less pronounced compared with structurally and histologically normal tissues (Control_His) (see Supplementary data online, *Figure S17* and *Tables S17* and *S18*, https://cardiogene.shinyapps.io/spatial_cmp/). This finding aligns with clinical expectations, as patients with end-stage HF, who have minimal normal tissue available for compensation, are often refractory to treatment.

## Discussion

Our primary objective in this study is to create a clinically relevant resource by offering gene expression profiles of cardiomyopathy in targeted cell types within regions characterized by distinct histological features, from a diverse and substantial patient cohort. Initially, we explored cellular composition and gene expression profiles in segments enriched with marker antibodies in cardiac tissue, identifying representative genes and pathways in targeted cells. Cell deconvolution revealed distinct cell composition, with more cardiomyocytes and fewer fibroblasts in the normal ventricular myocardium compared with previously reported sc/snRNA-seq analyses using transmural tissue. It validated the GeoMx platform’s compartmentalization efficiency, which was high for cardiomyocytes but modest for endothelial cells and fibroblasts. Secondly, we found that gene regulation in HF is influenced by cellular context, showing contrasting patterns across cell types, underscoring the limitations of traditional bulk transcriptional profiling. Third, we identified differentially expressed genes linked to specific histologic features, such as fibrosis, hypertrophy, and degeneration. Key genes, including *UCHL1, IGFBP2, CCL14, ACKR1,* and *PLVAP*, were validated through immunohistochemistry. Multiplex immunohistochemistry and integrative analysis of previous sc/snRNA-seq data uncovered *PLVAP, ACKR1, and CCL14*-positive pro-inflammatory endothelial cell subtype associated with fibrosis in HF. Consistent up-regulation of inflammation-related genes in endothelial cells across all histologic features suggests a role for vascular inflammation and endothelial activation in HF progression. Additionally, downregulation of ribosomal proteins in cardiomyocytes associated with myocyte disarray in HCMpEF highlights a potential research avenue. Lastly, by categorizing tissue segments based on histology and clinical features, we identified molecular profiles associated with various states of HF progression. Reciprocal regulation between compensated and uncompensated HF states highlighted genes potentially linked to HF development and progression. Among these, rare variant analysis in the UK Biobank identified *TAX1BP3, CRIP3*, and *PFKFB2* as associated with HF. These findings suggest that molecular alterations between different HF states may reveal processes underlying the progression from compensated to uncompensated HF. Gene expression profiles and GSEA can be interactively explored at https://cardiogene.shinyapps.io/spatial_cmp/.

### Advantage of integrating histologic features with clinical phenotypes

A major advantage of spatial transcriptomics is its ability to seamlessly integrate histologic features into gene expression profiling, allowing the analysis of gene expression in HF based on both histologic features and cell types, surpassing the limitations of technologies like sc/snRNA-seq. To evaluate the additional insights provided by histology-based analysis, we assessed the correlation between gene expression variation explained by clinical phenotypes and histologic features (see Supplementary data online, *Figure S11*). In cardiomyocytes, we observed a weak or negative correlation, indicating that clinical phenotypes and histologic features independently influence gene expression. For endothelial cells, a slight positive correlation was noted, although it remained lower than correlations found among histologic features alone.

A key example of the strength of histology-based analysis is the identification of an endothelial cell cluster co-expressing *ACKR1, PLVAP*, and *CCL14,* strongly associated with fibrosis in failing hearts. This association was identified using spatial transcriptomics and further validated through immunohistochemistry when analysed based on histologic features. However, this significant relationship was not observed in previous snRNA-seq analyses or in our immunohistochemistry validation when clinical phenotypes, rather than histologic features, were used as the basis for analysis.

Another significant advantage of histology-based analysis is its ability to differentiate structurally and histologically preserved myocardium under stress (‘compensated’) from advanced, morphologically abnormal myocardium (‘uncompensated’). By categorizing HF tissue segments into these states, we identified genes with reciprocal regulation, highlighting their roles in HF progression. For example, oxidative phosphorylation and myogenesis were up-regulated in histologically normal but diseased hearts compared with clinically and histologically normal hearts and were down-regulated in end-stage HF compared with compensated hearts, suggesting their role as compensatory mechanisms. Building on this reciprocal regulation, we could identify significant HF-associated genes, including *TAX1BP3, CRIP3*, and *PFKFB2*.

One notable advantage of histology-based analysis, as demonstrated in this study, is its ability to reduce variability in features such as treatment regimens and comorbidities. Grouping by clinical diagnoses often introduces substantial variability in pharmacologic treatments and comorbidities across comparisons (see Supplementary data online, *Tables S7* and *S8*), making it challenging to differentiate between treatment-induced gene expression changes and those caused by the disease itself. In contrast, histology-based analyses result in more balanced treatment regimens and comorbidities across comparisons (see Supplementary data online, *Tables S10* and *S11*), likely because clinical phenotypes are redistributed according to histologic features.

### Differential TNFα signalling across cell types: implications for targeted HF therapies

The complex regulation of inflammation-related gene sets by cell types and disease status also highlights the significance of considering both cell types and histology. Inflammation has been blamed for the initiation and progression of HF, but anti-inflammatory and immune-modulatory therapies have yielded ambiguous results in clinical trials.^67^ For example, the pro-inflammatory cytokine TNFα was elevated in HF patients,^68^ had detrimental effects on heart function pre-clinically,^69,70^ and thereby was considered a promising therapeutic target.^71,72^ However, randomized placebo-controlled trials showed no advantage of targeting TNFα in patients with HF or even adverse effects at higher doses.^71^ Our analysis may shed light on this outcome and suggest a workaround. Here, the TNFα gene set was down-regulated in cardiomyocytes while up-regulated in endothelial cells when comparing DCM and ICM with control. While TNFα signalling was up-regulated in cardiomyocytes when comparing Diseased_ES and Control_His, it remained down-regulated relative to Control_Clin, suggesting the activation of TNFα signals is still below physiologic level in cardiomyocytes of end-stage HF. This refutes the relevance of the TNFα signal as a therapeutic target in cardiomyocytes. However, in endothelial cells, the TNFα gene set was activated across both clinical and histologic comparisons (see Supplementary data online, *Figure S15B*), supporting its potential as a therapeutic target in this cell type. This suggests that targeting TNFα in endothelial cells rather than broadly inhibiting TNFα could be more effective, warranting further investigation.

### Overview of spatial transcriptomic approaches and rationale for GeoMx selection in cardiomyopathy analysis

Spatial transcriptomics technologies are advancing rapidly and diversely. Among the commercially popular methods, Visium and GeoMx WTA stand out.^28,73^ Visium uses a pre-defined grid of spots on slides to capture polyadenylated RNA, enabling comprehensive RNA visualization across tissue sections. This is valuable for a broad view of tissue architecture and cellular interactions,^74^ but its ROI selection is limited to pre-defined grids, restricting dynamic adjustments. In contrast, the GeoMx Human WTA uses antibodies and RNA probes with photocleavable oligonucleotide barcodes to quantify over 18 000 genes.^29,75^ It provides flexibility for dynamically selecting ROIs based on histological features and fluorescent markers, allowing targeted analysis of specific cell types or tissue structures. However, the amount of RNA data obtainable from a single slide remains limited, while recent advancements enable near single-cell resolution. Other technologies, like CosMx and MERFISH, achieve high-resolution profiling at the single-cell or subcellular level, unraveling the complex cellular and molecular interactions.^28^ However, their application in broad-scale studies is constrained due to low throughput and limited coverage. Balancing resolution, throughput, and cost is essential when selecting a spatial transcriptomics method suited to a study’s objectives. For our study, which focuses on gene expression in cardiac cell types across different histology in diverse cardiomyopathies and involves a substantial number of patients, the GeoMx platform offers the optimal balance of flexibility, segmentation, and clinical relevance over single-cell resolution.

### Study limitation

Our study demonstrated that while the GeoMx platform offers effective compartmentalization, particularly for cardiomyocytes, it presents some challenges in accurately segmenting densely packed or intertwined cell types such as endothelial cells and fibroblasts. Limitations in antibody specificity, including the overlap of CD31 and Vimentin with other cell types, can lead to unintended non-specific staining. Additionally, the platform’s restriction to three antibodies limits comprehensive visualization of all cell types or subtypes. These challenges underscore the need for careful marker selection and interpretation when using GeoMx in complex tissue environments. Furthermore, the requirement for a minimum cell count on the GeoMx platform constrained our ability to compare fibroblasts between normal and failing hearts. Recent advancements have enabled near single-cell resolution for improved precision but reduce tissue coverage, throughput, and cost efficiency, highlighting challenges in balancing detailed analysis with broader tissue surveys. While differences in comorbidities and treatments across clinical groups could pose a limitation by introducing variability (see Supplementary data online, *Tables S7* and *S8*), our approach of utilizing histologic features and integrating histologic data with clinical phenotypes ensured a better balance between groups (see Supplementary data online, *Tables S10, S11, S13*, and *S15*). Furthermore, in differential gene expression analyses where comorbidities and treatments were included as covariates, the log fold change (logFC) values showed a strong correlation with those from the original analysis, further supporting the robustness of our findings (see Supplementary data online, *Figure S18*). To focus on the intrinsic myocardial properties at various stages of heart failure and to minimize secondary hemodynamic effects or biases associated with more uniform controls, we included LV samples with normal contractility and histology, even in cases of RV failure or elevated BNP levels. While this approach increases the variability of controls and may help reduce non-specific findings, it could also decrease sensitivity in detecting differences in gene expression. Lastly, the number of tissue cores and cellular segments analysed was limited due to technical and logistical constraints such as batch effects, costs, and the availability of high-quality tissue samples. Future studies with expanded sample sizes could focus on specific disease types and histological characteristics, building on the foundational insights provided in this study.

### Conclusion

This study provides a transcriptional landscape in cardiomyocytes and endothelial cells from cardiomyopathies dissected by clinical and histologic features. We identified that gene expression profiles in cardiomyopathy differ by cell type and considering histologic features can unveil potential pathophysiological mechanisms underlying progressive failing heart. This will further inform the pathways and potential therapeutic targets for HF.

## Supplementary Material

ehaf272_Supplementary_Data

## Declarations

### Data Availability

The data associated with this study has been submitted to the GEO repository under the accession number GSE271676 and will be publicly available upon official publication of the paper. Additionally, the data and accompanying code can be accessed through the following link: Mendeley Data. Further information and requests for resources can be directed to the lead contact, Sang Eun Lee, at sangeunlee.md@amc.seoul.kr / sangeunlee.md@gmail.com.
