# Supplementary material for: Spatial transcriptional landscape of human heart failure
Source: Eur Heart J. 2025 May 8;46(31):3098–114. doi: 10.1093/eurheartj/ehaf272 (PMC12349961; doi:10.1093/eurheartj/ehaf272)
Supplement: ehaf272_Supplementary_Data [file ehaf272_supplementary_data.zip › SupplementaryFigures_EHJ2ndrevision_20250210.docx]

**Supplementary Fig.1**

**
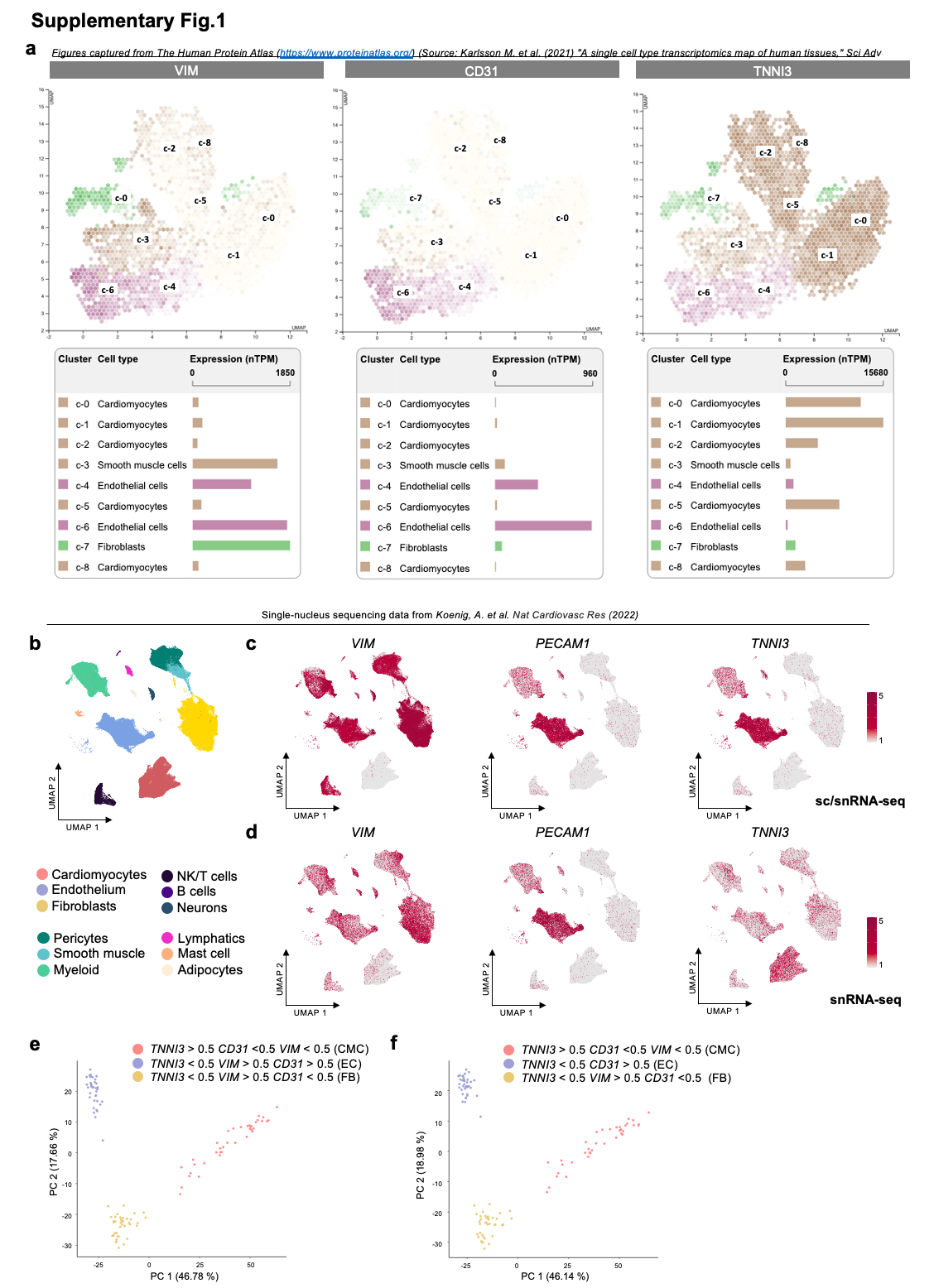
Supplementary Fig.1 Marker specificity validation with external single-cell/single-nucleus RNA sequencing data a.** Figures captured from The Human Protein Atlas (https://www.proteinatlas.org/) with minor modification for better readability. hUMAP plot representing single-cell RNA sequencing data from heart tissue, highlighting the expression levels of key marker genes (Troponin I (TNNI3), CD31, and Vimentin (VIM)) in cardiomyocytes, fibroblasts and endothelial cells of the heart muscle. Different colors represent distinct cell populations: purple for endothelial cells (c4, c6), green for fibroblasts (c7), brown for cardiomyocytes (c0, c1, c2, c5, c8), and beige for smooth muscle cells (c3). The intensity of the color indicates expression levels measured in normalized transcript per million (nTPM). Source: Karlsson M. et al. (2021) "A single cell type transcriptomics map of human tissues," Sci Adv, 28;7(31): abh2169. Available at: The Human Protein Atlas (<https://www.proteinatlas.org/>). **b.** UMAP plot showing the clustering of sc/snRNA-seq from heart tissue (reanalysis using original source Koenig et al. 2022), colored by cell type. Different colors represent distinct cell populations identified in the study, including cardiomyocytes, endothelial cells, fibroblasts, and other cell types. **c-d.** UMAP plots highlighting the expression levels of key marker genes (TNNI3, CD31, and VIM) in sc/snRNA-seq (**c**) and snRNA-seq (**d**) of heart tissue. The color indicates the normalized expression of the marker genes**. e-f.** PCA plot illustrating pseudo-bulk analysis at the patient level, aggregating single nuclei based on the normalized expression levels of marker genes to emulate our antibody-based cell selection strategy in snRNA-seq: **e.** Aggregated groups of cells selected as endothelial cells (EC) based on VIM >0.5 and CD31 >0.5 expression levels. **f.** Aggregated groups of cells selected as endothelial cells (EC) based on CD31 >0.5 expression levels only. Red dots represent cardiomyocyte (CMC) AOIs, purple dots represent endothelial cell (EC) AOIs, and yellow dots represent fibroblast (FB) AOIs. Total number of subjects included is 38, and the total number of single cell and nuclei included is 48,556 and 203,333, respectively.

**Supplementary Fig.2**


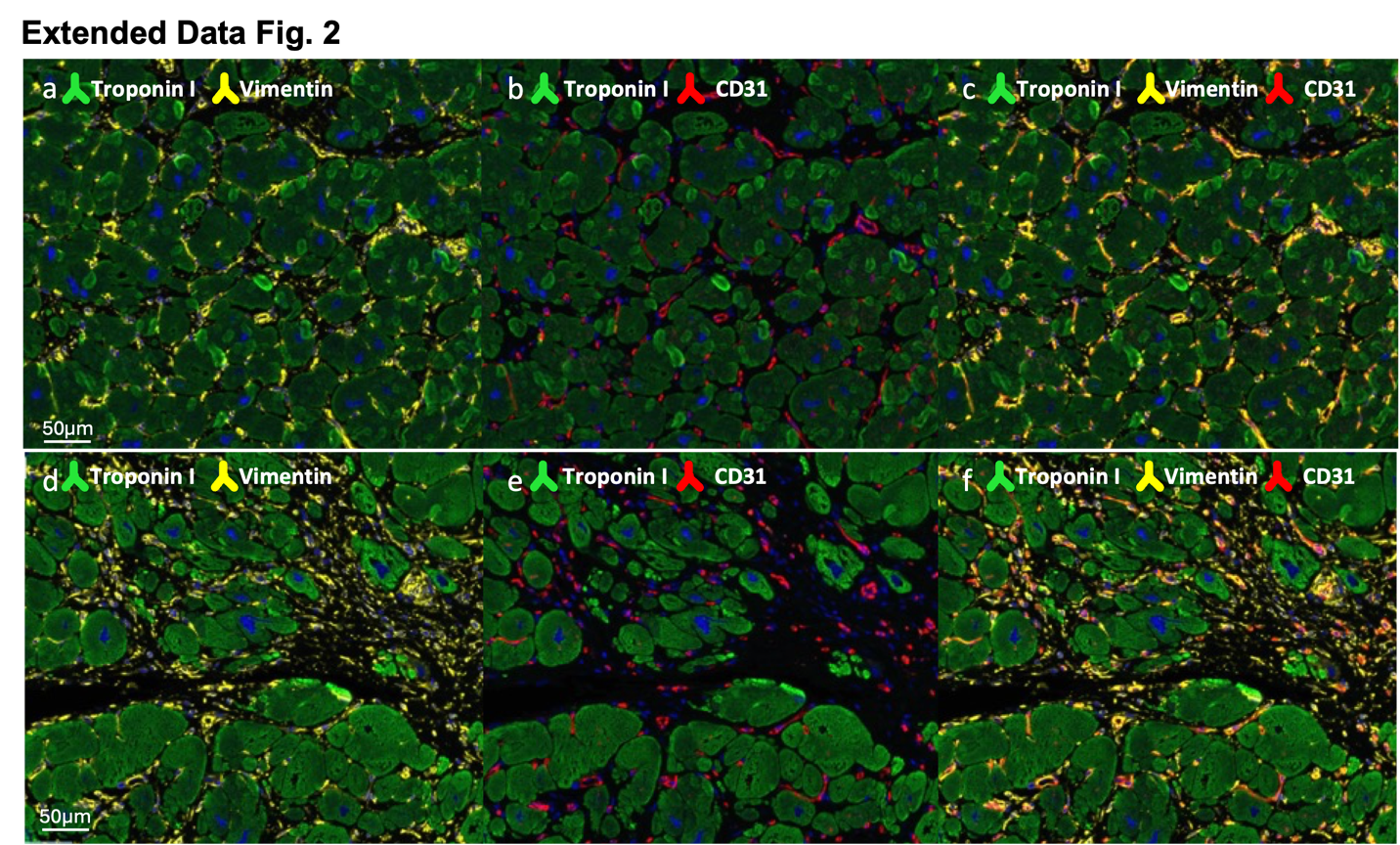


**Supplementary Fig.2.** **Validation of antibody staining for target cells a-f.** Immunofluorescence staining images showing representative regions of interest (ROI) used in our GeoMx experiment. Panels (**a-c**) depict control ROI, while panels (**d-f**) depict ROI with fibrosis. Immunofluorescence staining for Vimentin (yellow, **a,d**), CD31 (red, **b,e**), Troponin I (green), and DAPI (blue) and co-localization of Vimentin and CD31 in endothelial cells (**c, f**). The scale bar represents 50μm.

**Supplementary Fig.2 (continued)**


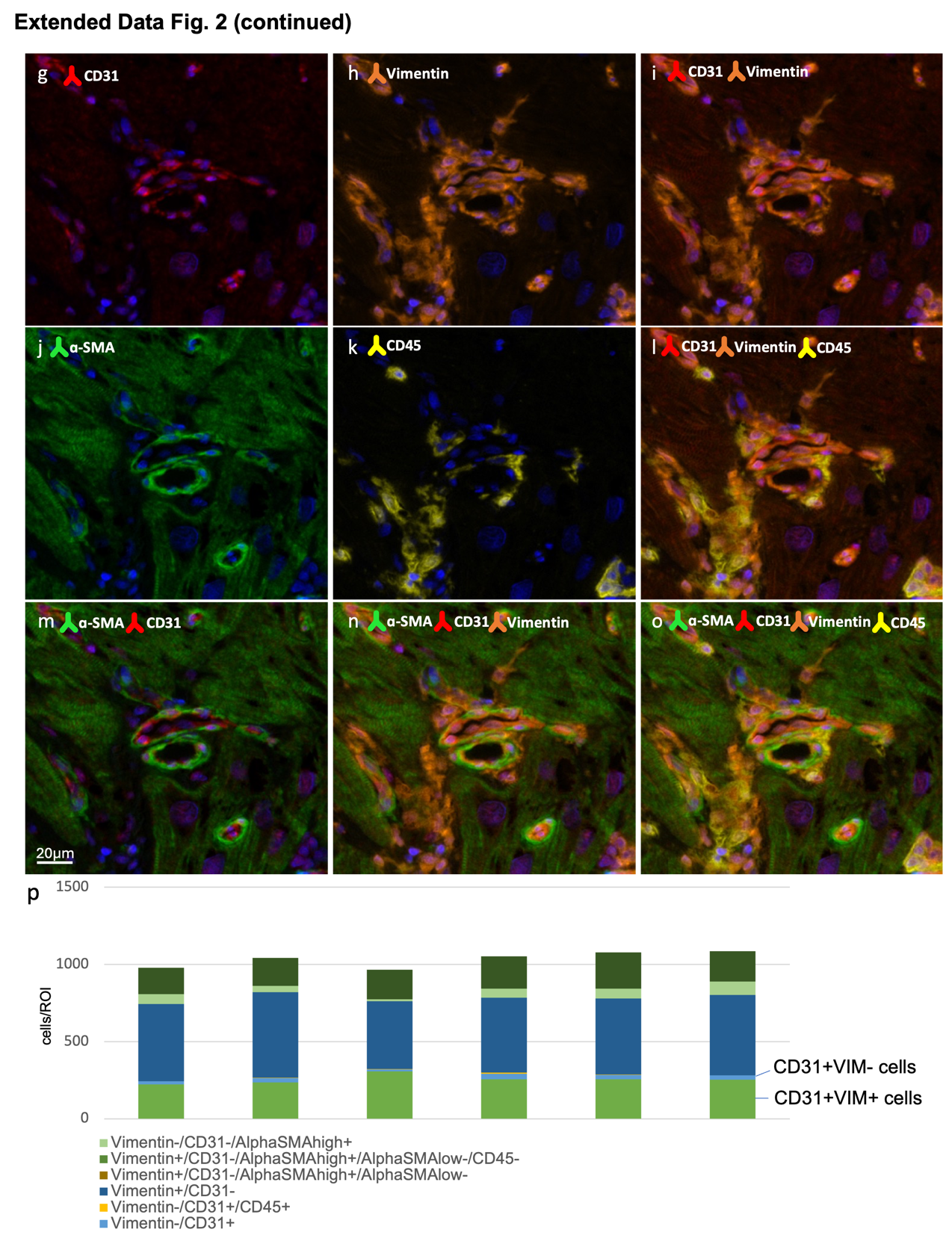


**Supplementary Fig.2 (continued)** **Validation of antibody staining for target cells g-o.** Multiplex immunohistochemistry staining showing DAPI (blue), CD31 (red, **g**), Vimentin (orange, **h**), colocalization of Vimentin and CD31 (**i**), alpha-SMA (green, **j**), CD45 (yellow, **k**), colocalization of Vimentin, CD31 and CD45 (**l**), colocalization of CD31 and alpha-SMA (**m**), colocalization of alpha-SMA, Vimentin, and CD31 (**n**), and colocalization of all markers (**o**). This demonstrates the co-localization of Vimentin and CD31 in endothelial cells, and challenges in cell segmentation due to the intertwined structure of cells. **p.** Bar graph depicting the automated cell counts of distinct cell types across five ROIs in a tissue sample from the subject labeled AMC008. This reveals that the majority of CD31-positive endothelial cells were also Vimentin-positive (91±3%).

**Supplementary Fig.3**


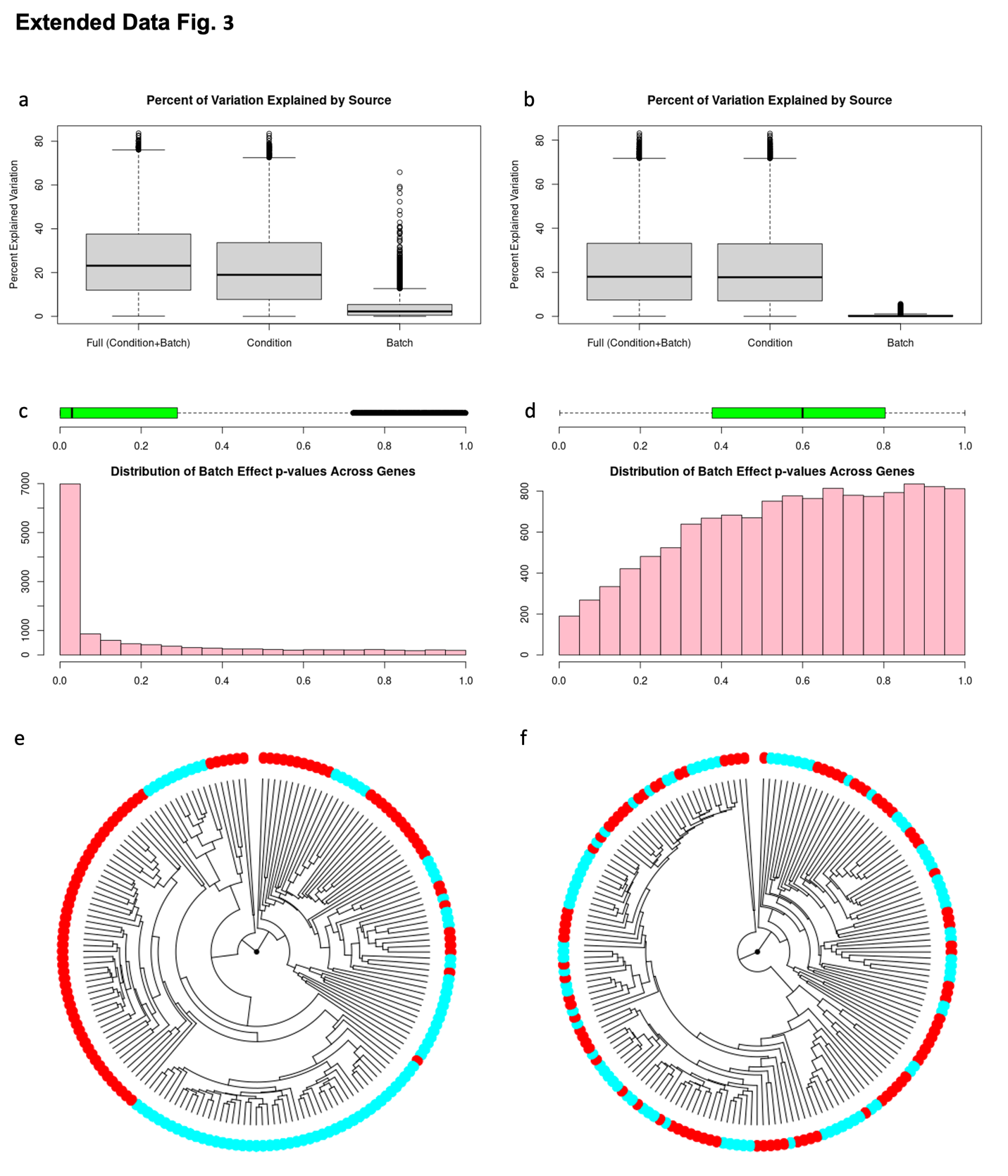


**Supplementary Fig.3.** **Batch effect correction.** Percent of variation explained by condition (cell types) and batch (slides or TMA blocks) before **(a)** and after **(b)** batch correction with ComBat-seq. Distribution of batch effect p-values across genes before **(c)** and after **(d)** batch correction. Circular dendrogram showing sample clustering before **(e)** and after **(f)** batch correction (red and blue indicate different batches). (TMA, tissue microarray) (n=178)

**Supplementary Fig.4**


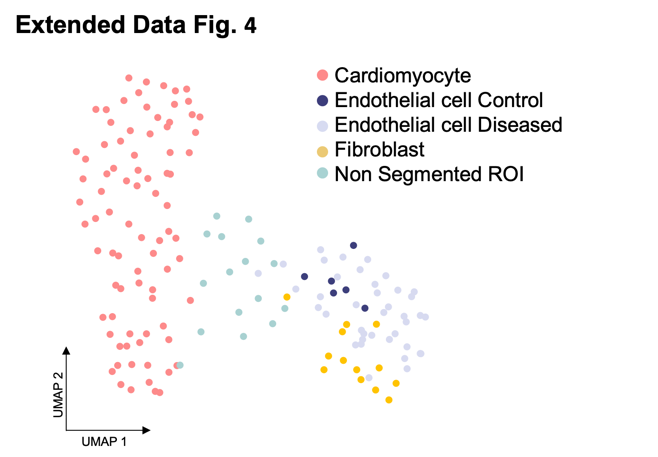


**Supplementary Fig.4.** **UMAP visualization of segments highlighting endothelial cells by disease status** UMAP representation of each segment (Area of Interest, AOI) and non-segmented regions, with distinct colors representing different cell types. Endothelial cell segments from control subjects are colored dark blue, while endothelial cell segments from cardiomyopathy patients are colored light purple. (n=157)

**Supplementary Fig.5**


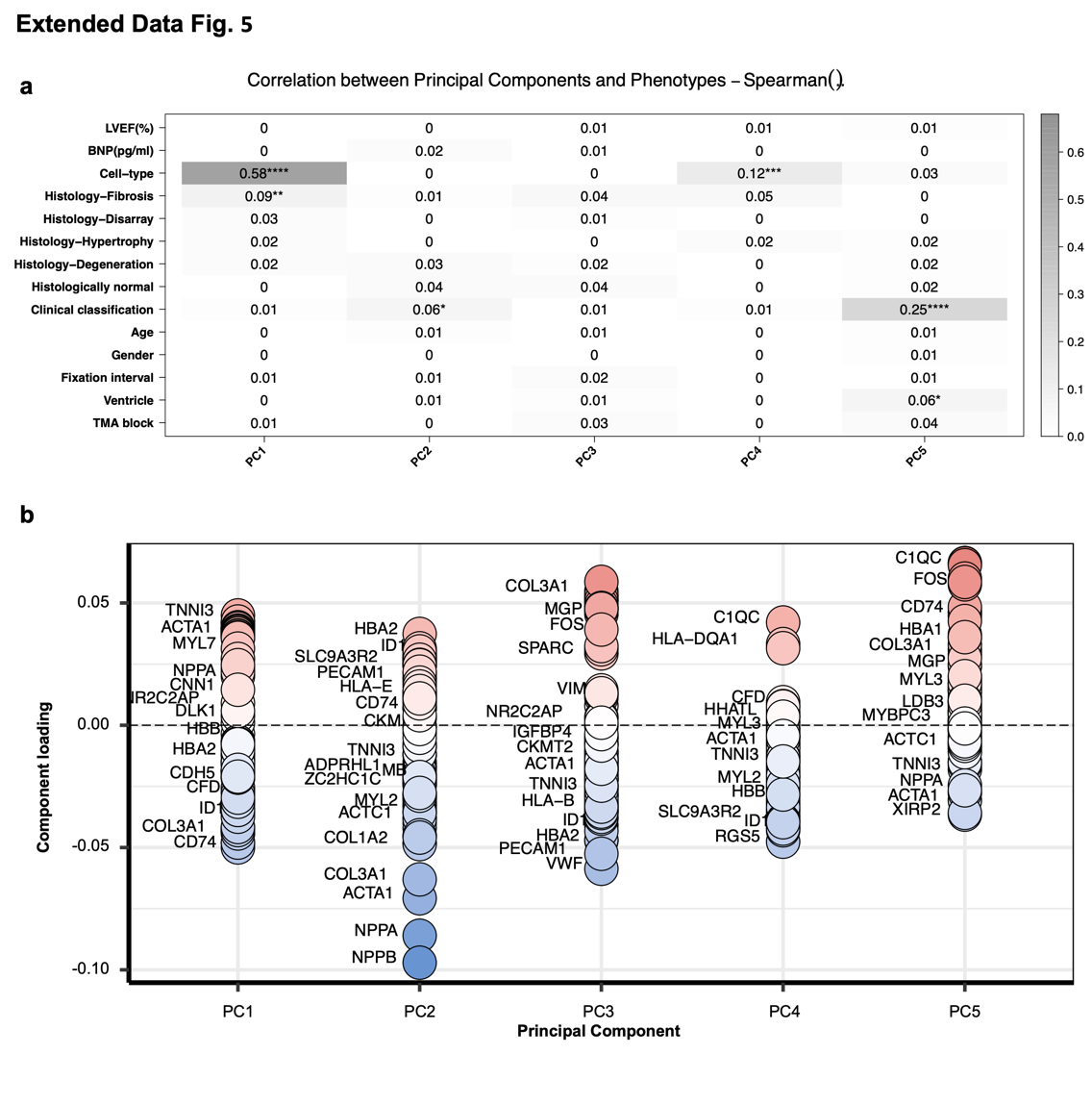


**Supplementary Fig.5.** **Principal components analysis a.** The table presents the Spearman rank correlation coefficients (ρ) between Principal Components (PCs) and clinical variables. Significance levels are indicated as follows: (****, p-value <0.0001; ***, p-value <0.001; **, p-value <0.01; *, p-value <0.05). (LVEF, left ventricular ejection fraction; BNP, brain natriuretic peptide level; TMA, tissue microarray). **b**. Genes with positive and negative component loading for 5 PCs. (n=157)

**Supplementary Fig.6**

**Supplementary Fig.6.** **Proportions of cell types based on clinical conditions and histology a.** Donut plots illustrating the proportion of cell types across various clinical phenotypes on non-segmented regions of interest (ROIs), calculated by SpatialDecon^1^. Each donut plot represents a different clinical condition, showing the relative abundance of various cell types as indicated by the color legend. Panels **b-e** show donut plots illustrating the proportion of cell types by grades of histology on non-segmented ROIs, calculated by SpatialDecon. Panel **b** represents hypertrophy, panel **c** represents degeneration, panel **d** represents fibrosis, and panel **e** represents disarray. The numbers on each plot indicate the percentage ± standard deviation of each cell type. The number in parentheses indicates the sample number. The statistical significance was calculated with Kruskal-Wallis test for each cell-type. (DCM, dilated cardiomyopathy; ICM, ischemic cardiomyopathy; HCMpEF, hypertrophic cardiomyopathy with preserved ejection fraction; HCMrEF, hypertrophic cardiomyopathy with reduced ejection fraction.)

**Supplementary Fig.7**

**Supplementary Fig.7.** **Differentially expressed genes and clustering across target cell-types a.** Dot plot depicting upregulated and down-regulated genes across various cell types. Each lane represents a different cell type comparison. Five genes with the lowest p-values are annotated in each lane. Each dot, colored specific to cell type as indicated in the legend (upper right panel), represents a positively differentially expressed gene. The numbers above each lane indicate the counts of upregulated genes, while the numbers below each lane indicate the counts of downregulated genes for each comparison. (CMC: cardiomyocytes; EC: endothelial cells; FB: fibroblasts; No_Seg: non-segmentation cells; Oth: other segments). **b.** Heatmap based on top and bottom 100 differentially expressed genes across cell types, depicting clustering of segments according to cell types. Each row represents a gene, and each column represents a segment. The color scale represents logCPM (log-counts per million). (The total number of samples is 157, with CMC: 83, EC: 45, FB: 13, and No_Seg: 16)

**Supplementary Fig.8**


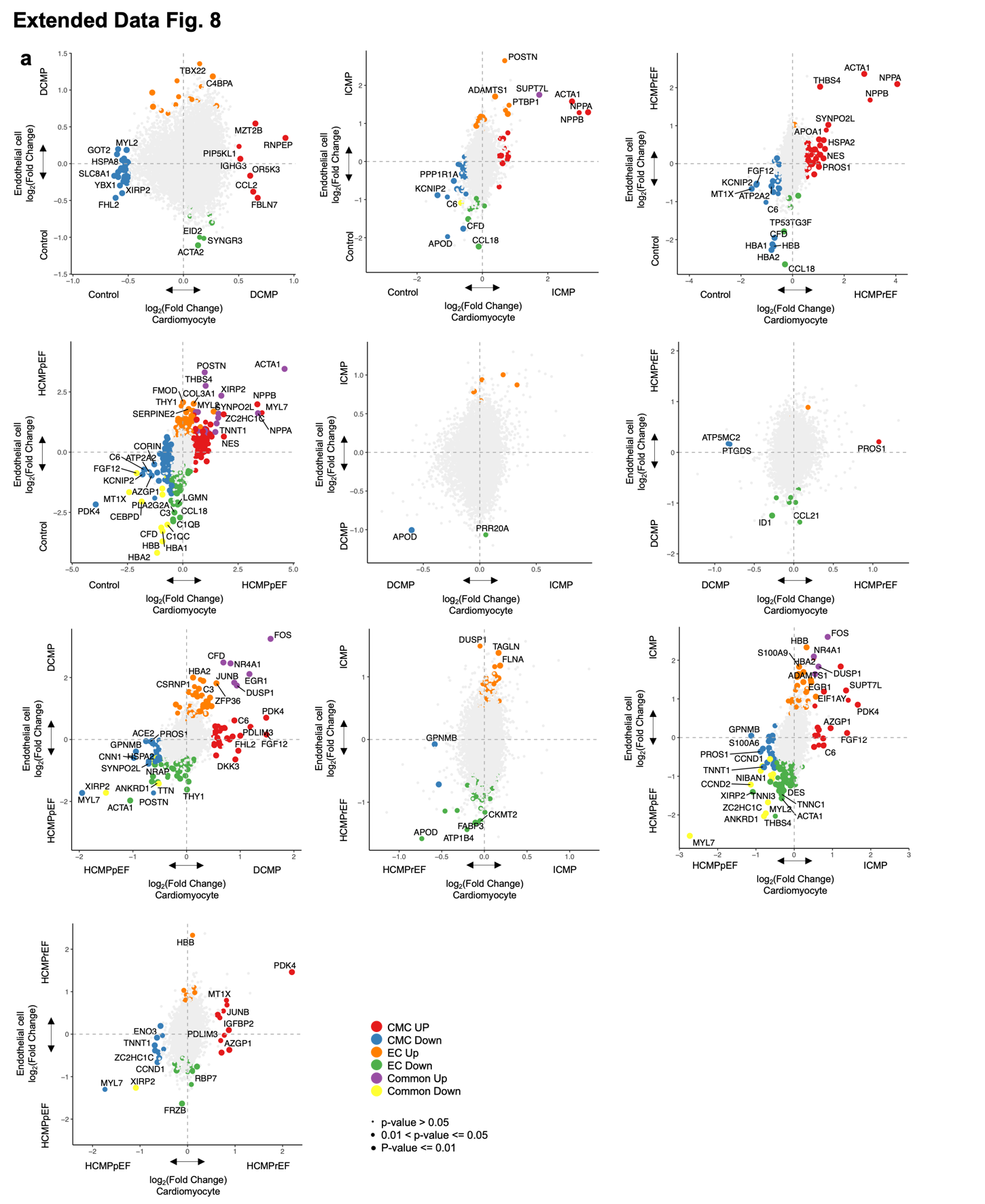


**Supplementary Fig.8**. **Comparison of differentially expressed genes in cardiomyocytes and endothelial cells across various cardiomyopathies** Scatter plots showing differentially expressed genes in either cardiomyocyte segmentation (x- or endothelial segmentation (y-axis) from the differential expression analysis between specific clinical conditions (Control, DCM, ICM, HCMrEF, HCMpEF). Each scatter plot compares two conditions within the specified cardiomyopathy. Red indicates genes significantly upregulated in CMC in one condition, blue indicates genes significantly upregulated in CMC in the other condition, orange indicates genes significantly upregulated in EC in the same condition as red in CMC, and green indicates genes significantly upregulated in EC in the same condition as blue in CMC. Purple and yellow indicate genes upregulated simultaneously in both CMC and EC in each condition. The size of the dots represents the adjusted p-value of DEGs, categorized into three groups based on thresholds of 0.05 and 0.01. CMC, cardiomyocyte; EC, endothelial cells; DCM, dilated cardiomyopathy; ICM, ischemic cardiomyopathy; HCMrEF, hypertrophic cardiomyopathy reduced ejection fraction; HCMpEF, hypertrophic cardiomyopathy preserved ejection fraction. The number of samples used in the comparisons for cardiomyocytes is: control (7), DCM (13-14), ICM (16), HCMrEF (11-13), and HCMpEF (18). For endothelial cells, the numbers are: control (6), DCM (8-10), ICM (7), HCMrEF (6-8), and HCMpEF (10). The number of samples varies due to the outlier selection process for each comparison, and the detailed number of samples used in each comparison is described in Supplemental Table 20.

**Supplementary Fig.9**
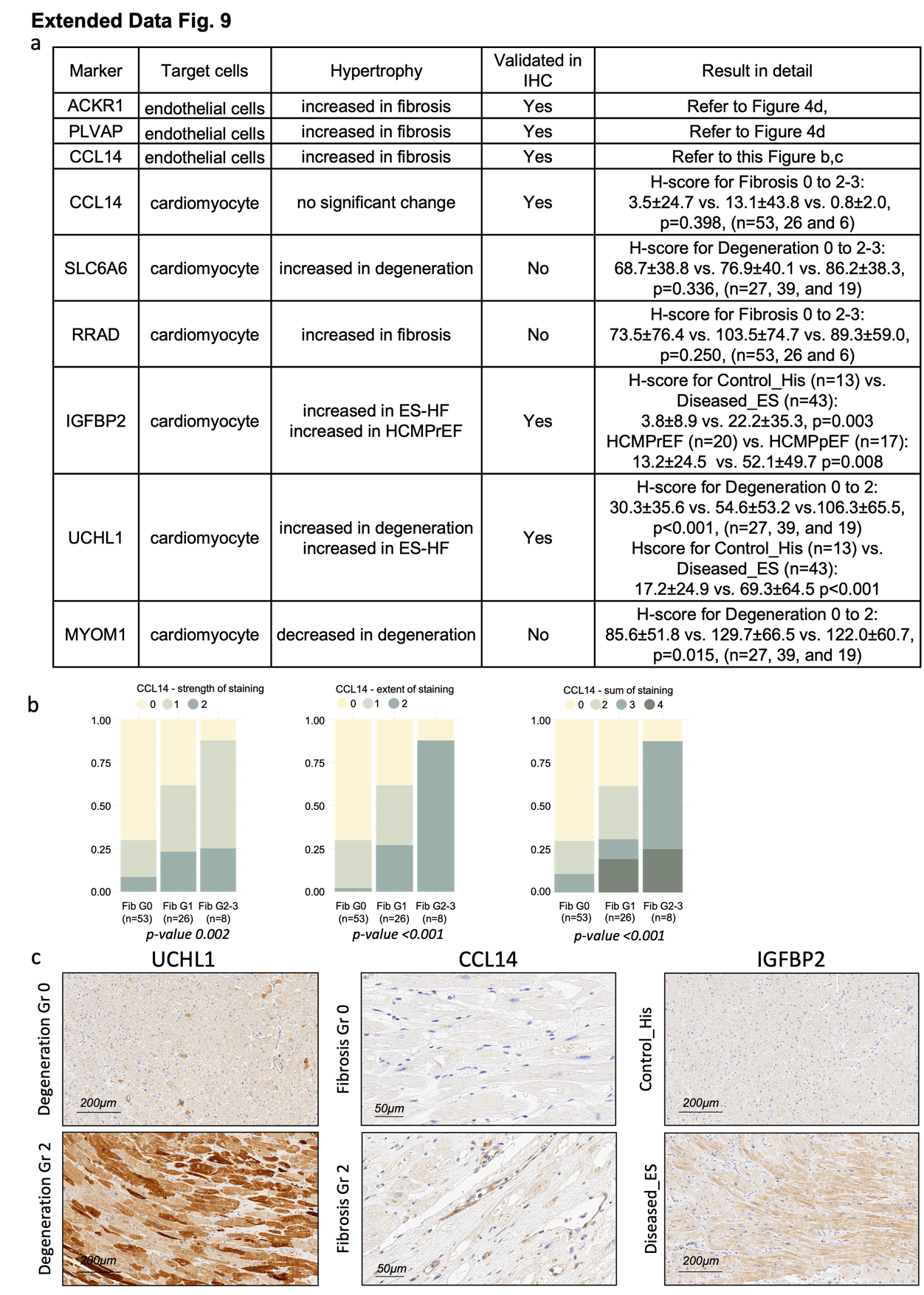


**Supplementary Fig. 9**. **Summary of immunohistochemistry staining for the validation of gene expression.** **a**. Summary table of genes tested. **b.** Bar graphs of CCL14 staining scores according to fibrosis grades. The left graph depicts the proportion of cores in Tissue microarray (TMA) blocks having each score of CCL14 staining strength, the graph in the middle indicates the scores of staining extents, and the right graph shows the sum of the two scores. P-values are from the Pearson's Chi-squared test. **c.** Representative figures of immunohistochemistry staining using UCHL1, CCL14, and IGFBP2 antibodies. The number of samples is described in the table or in parentheses.

**Supplementary Fig.10**


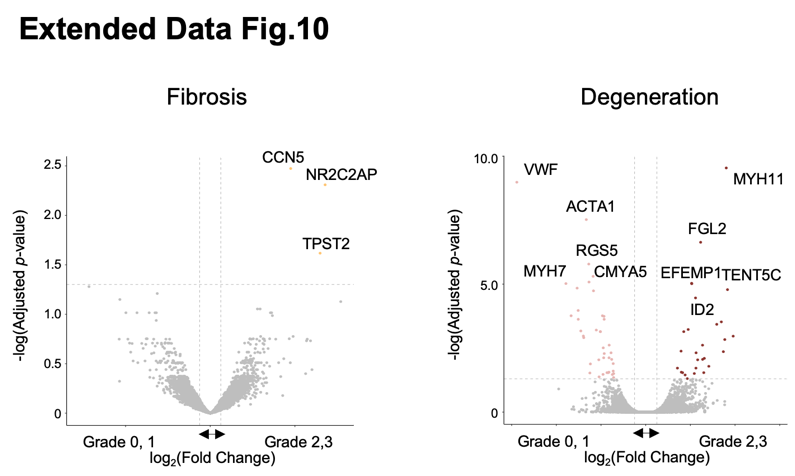


**Supplementary Fig.10 Differentially expressed genes in fibroblast according to histology** Volcano plot displaying the log fold change (logFC) and two-sided p-value from the differential expression analysis in fibroblast segments. The left panel shows the results for fibrosis, and the right panel shows the results for degeneration. Key genes with the lowest p-values are annotated in each plot. The number of samples used in the comparisons is as follows: Fibrosis Grade 0-1 (n=6) vs. Grade 2-3 (n=5), and Degeneration Grade 0-1 (n=5) vs. Grade 2-3 (n=6).

**Supplementary Fig.11**


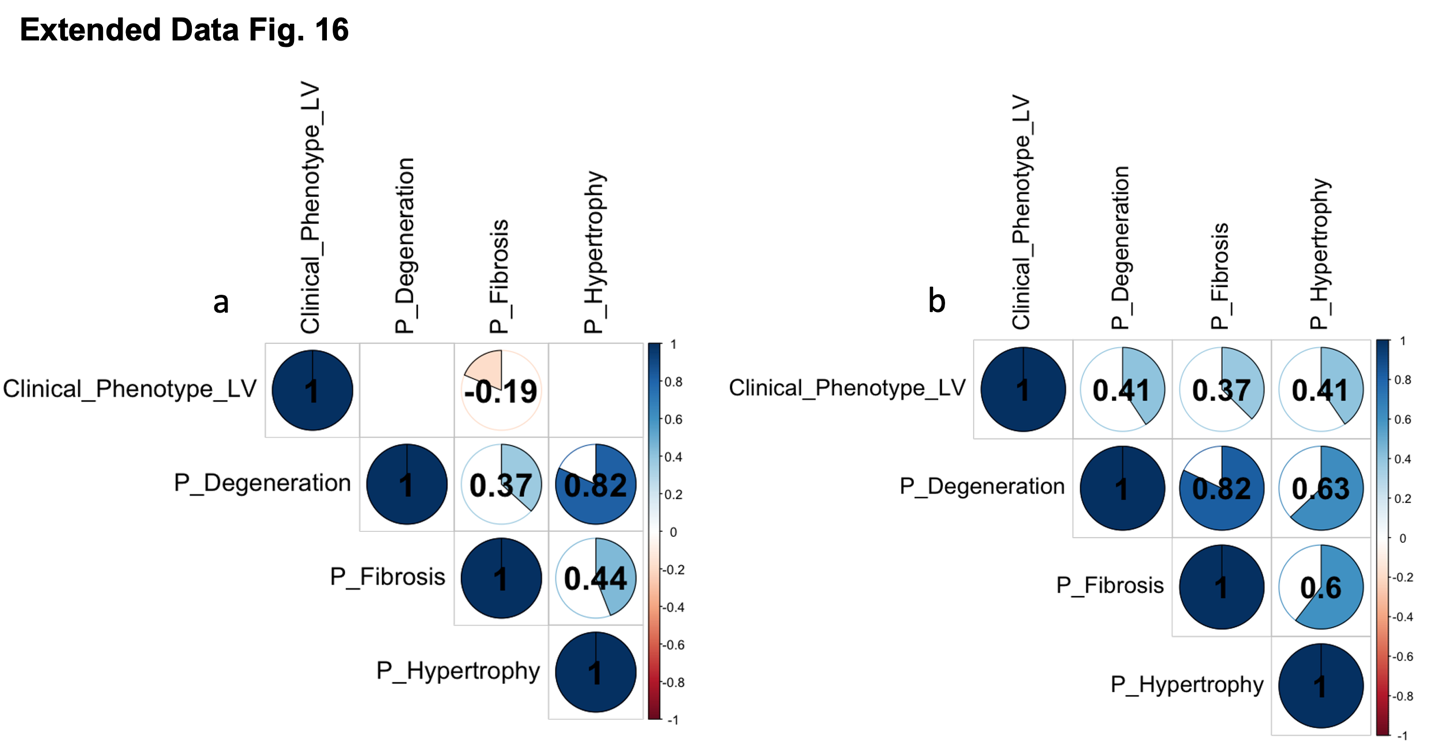


**Supplementary Fig.11.** **Correlation of gene expression variation explained by histological features and clinical diagnosis** Correlations of the amount of variation in gene expression explained by each histological feature and clinical diagnosis in either cardiomyocyte segmentation (**a**) or endothelial cell segmentation (**b**). The circle values indicate Pearson’s coefficients, and the blank boxes define insignificant correlations. The total sample size is 68 for cardiomyocytes and 39 for endothelial cells.

**Supplementary Fig.12**


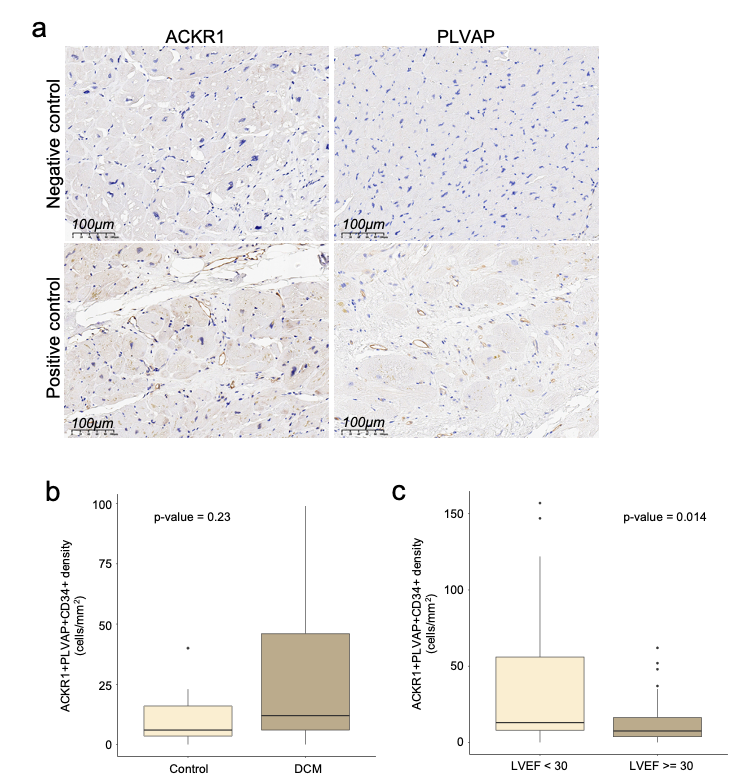


**Supplementary Fig.12**. **Positive and negative controls of immunohistochemistry staining of ACKR1 and PLVAP and cell densities from multiplex immunohistochemistry a.** Representative images of immunohistochemistry staining of cardiac tissue with ACKR1 (left) and PLVAP (right). The upper panels show negative controls, and the lower panels show positive controls. The scale bars indicate the magnification level. **b-c.** Boxplots representing the density of cells co-expressing CD34, ACKR1, and PLVAP, stratified by control vs. DCM and LVEF < 30% vs. LVEF ≥ 30%. For panel b, the sample numbers are 15 vs. 17. For panel c, the sample numbers are 37 vs. 43. Statistical significance was determined using a t-test.

**Supplementary Fig.13**


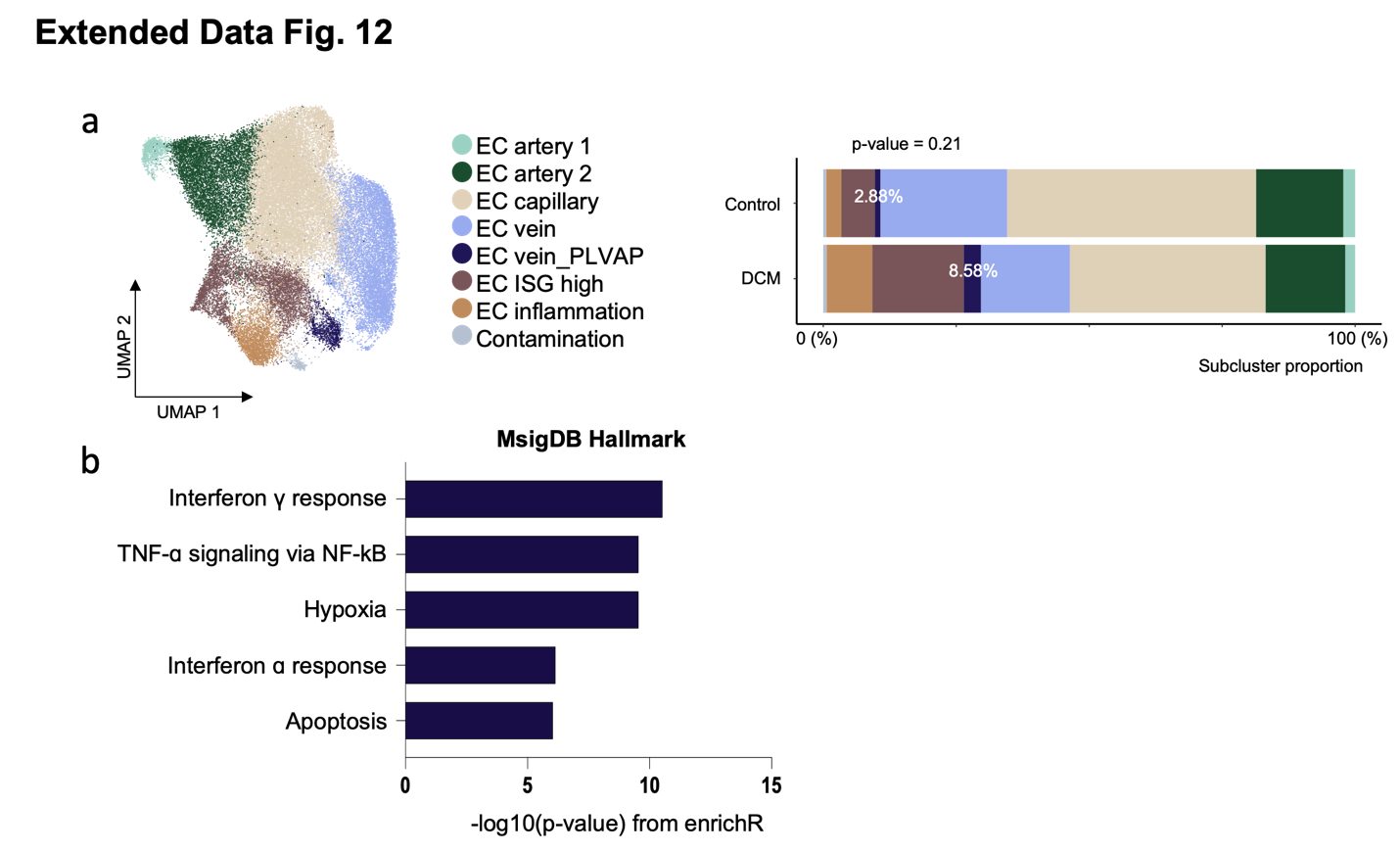


**Supplementary Fig.13.** **Proportions of endothelial subclusters and enrichment analysis** **a.** Endothelial subclusters on UMAP and their stacked bar plot showing the proportions of endothelial subclusters according to clinical phenotype from single-cell/single-nucleus RNA sequencing (sc/snRNA-seq) data of heart tissue, as conducted by Koenig et al. (2022). Each color represents a different subcluster, and the bar segments indicate the relative abundance of each subcluster within the specified clinical groups (y-axis). The sample numbers are 18 DCM and 27 control the total number of cells/nuclei included is 49,382. Statistical significance was determined using the Mann-Whitney test. **b.** Bar plots from enrichment analysis of marker genes for endothelial cell (EC) vein PLVAP depicting top 5 Hallmark gene set with -log10(p-values) using enrichR^2^. The height of each bar represents the significance level of the enrichment for each gene set. (MSigDB, The Molecular Signatures Database)

**Supplementary Fig.14**


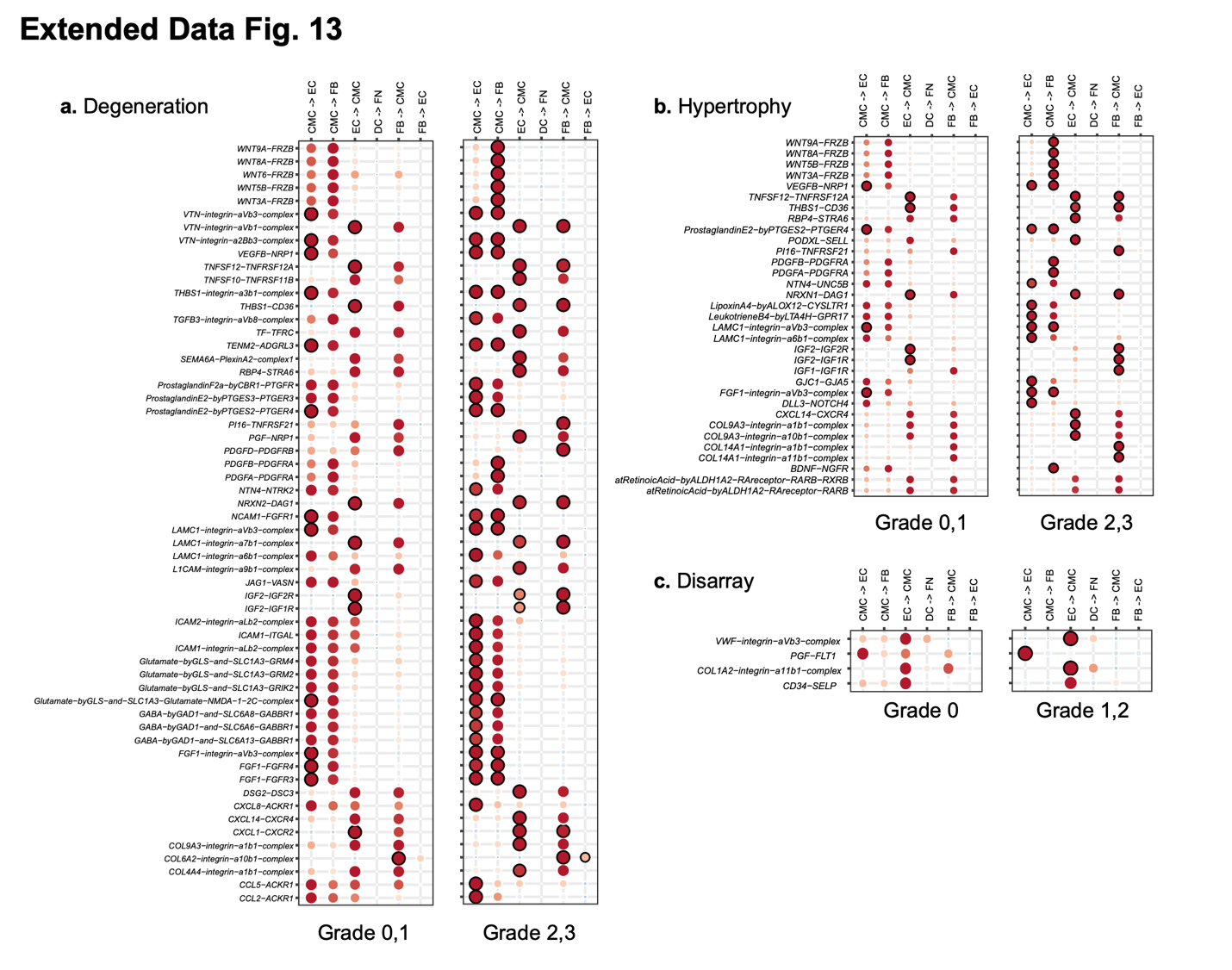


**Supplementary Fig.14. Segment-segment interaction analysis using CellphoneDB a-d.** The bubble dot plot displays interactions between cell types, analyzed using CellPhoneDB and visualized with R package ktplots. Comparisons are made across different histological conditions: degeneration (a), hypertrophy (b), disarray in HCMpEF (c), and fibrosis (d), grouped as Gr 0,1 versus Gr 2,3 (grade 0 versus 1-2 in disarray). **e.** Interactions related to ACKR1 and CCL14 are highlighted. The x-axis lists the interacting pairs, while the y-axis represents the different cell types. The color and size of the dots represent the strength of interactions, with significant interactions (p<0.05) outlined in red. HCMpEF, hypertrophic cardiomyopathy preserved ejection fraction. The sample numbers for each histological condition are as follows: degeneration (Grade 0,1: n=80; Grade 2,3: n=37), fibrosis (Grade 0,1: n=98; Grade 2,3: n=19), hypertrophy (Grade 0,1: n=105; Grade 2,3: n=12), and disarray (Grade 0: n=14; Grade 1,2: n=14).

**Supplementary Fig.14 (continued)**


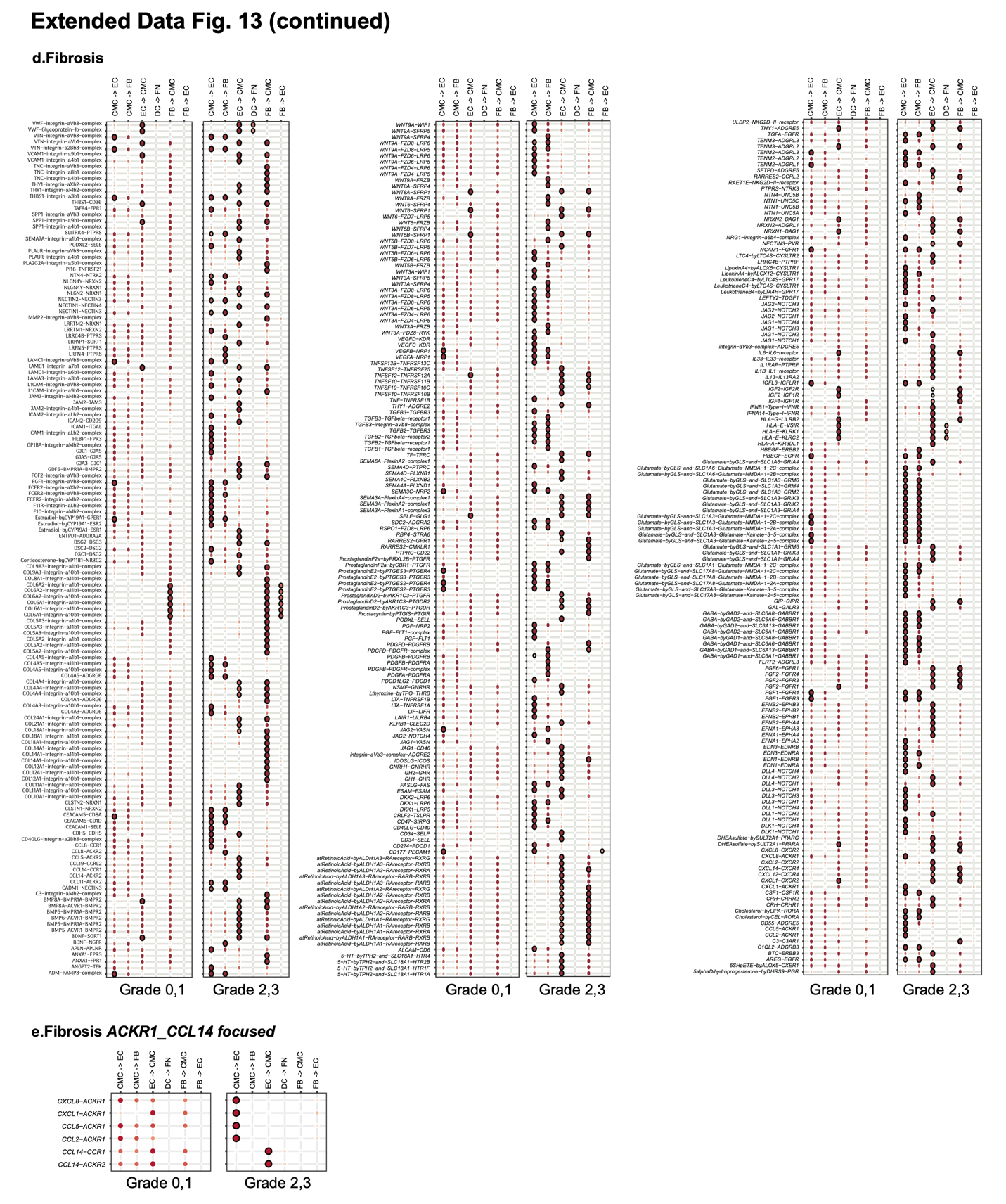


**Supplementary Fig.15**


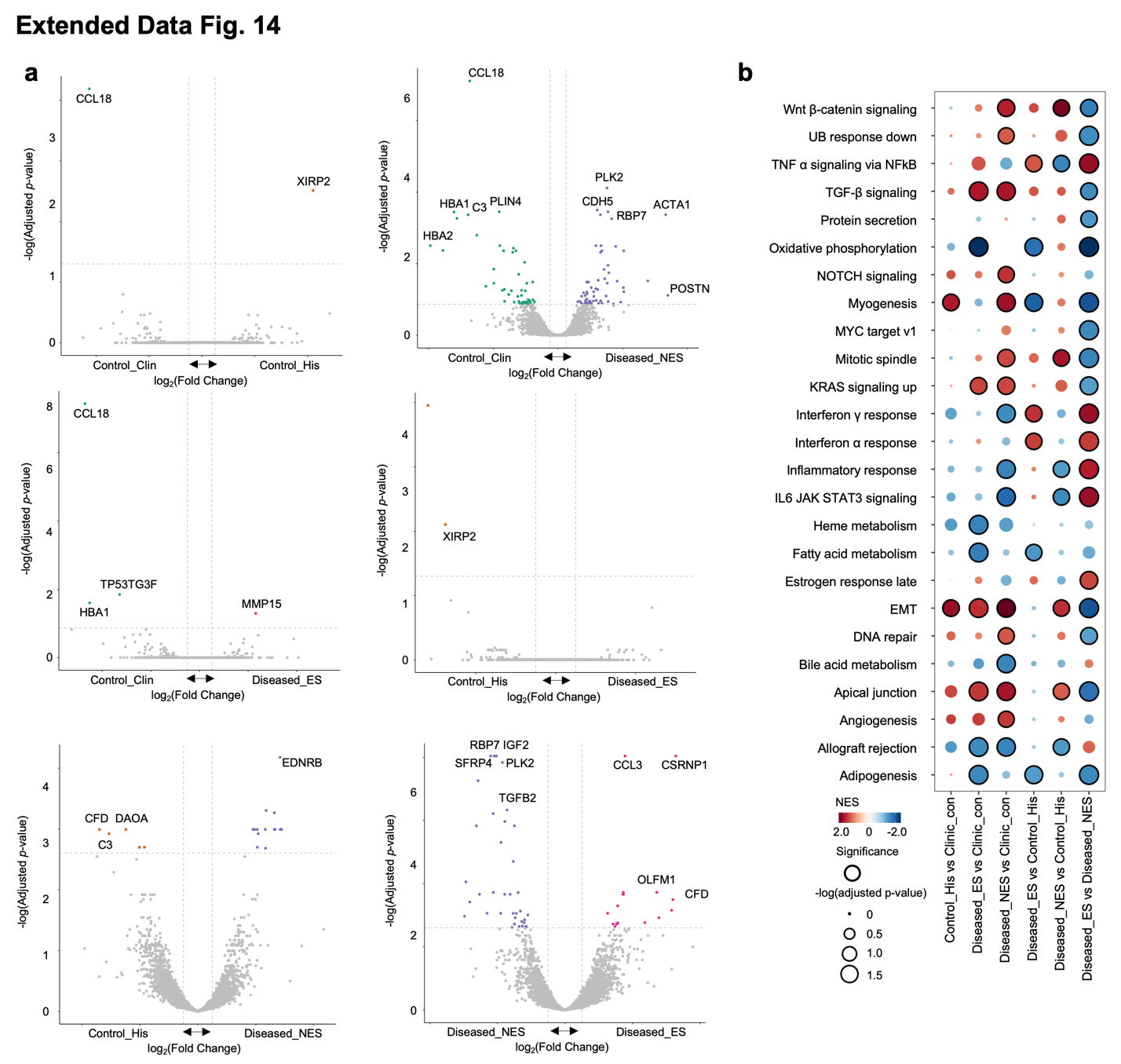


**Supplementary Fig.15. Differentially expressed genes and gene set enrichment analysis in endothelial cells integrating clinical and histologic features** **a.** Volcano plots displaying the log fold change (logFC) (x-axis) and two-sided p-value (y-axis) from the differential expression analysis in the comparison across different groups of endothelial cell segmentation. The color of the dots corresponds to the groups defined in Figure 5b, indicating genes that are significantly increased in each group. **b.** Dot plots depicting the Hallmark gene set for differential expression across the groups of endothelial segmentation. The size of the dots indicates the -log10 (adjusted p-value, Benjamini-Hochberg FDR), and the color reflects the NES score from gene set enrichment analysis (GSEA). The number of samples in each group for endothelial cells is as follows: Control_Clin (6), Control_His (5), Diseased_ES (20), and Diseased_NES (8).

**Supplementary Fig.16**


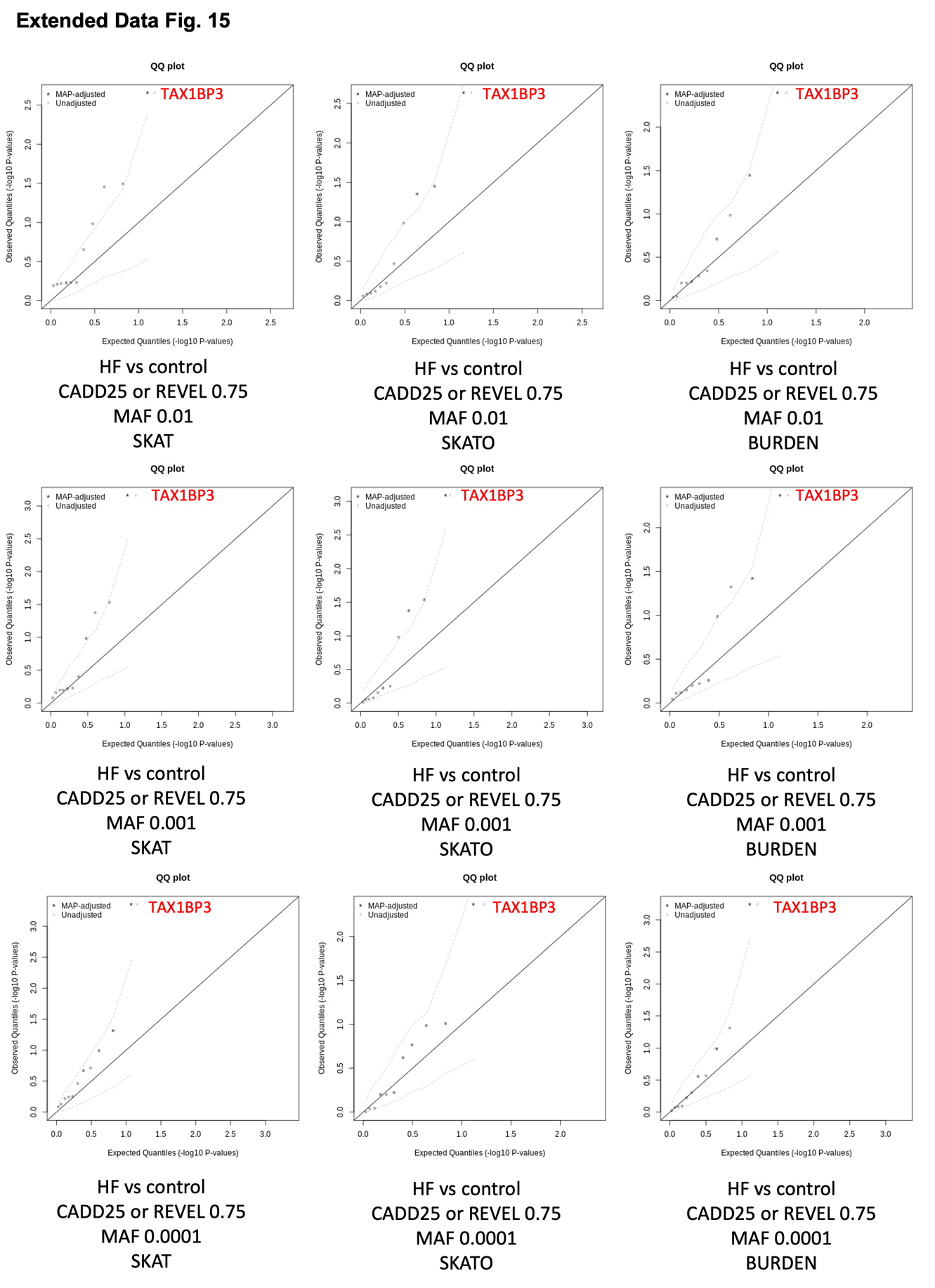


**Supplementary Fig.16 (continued)**


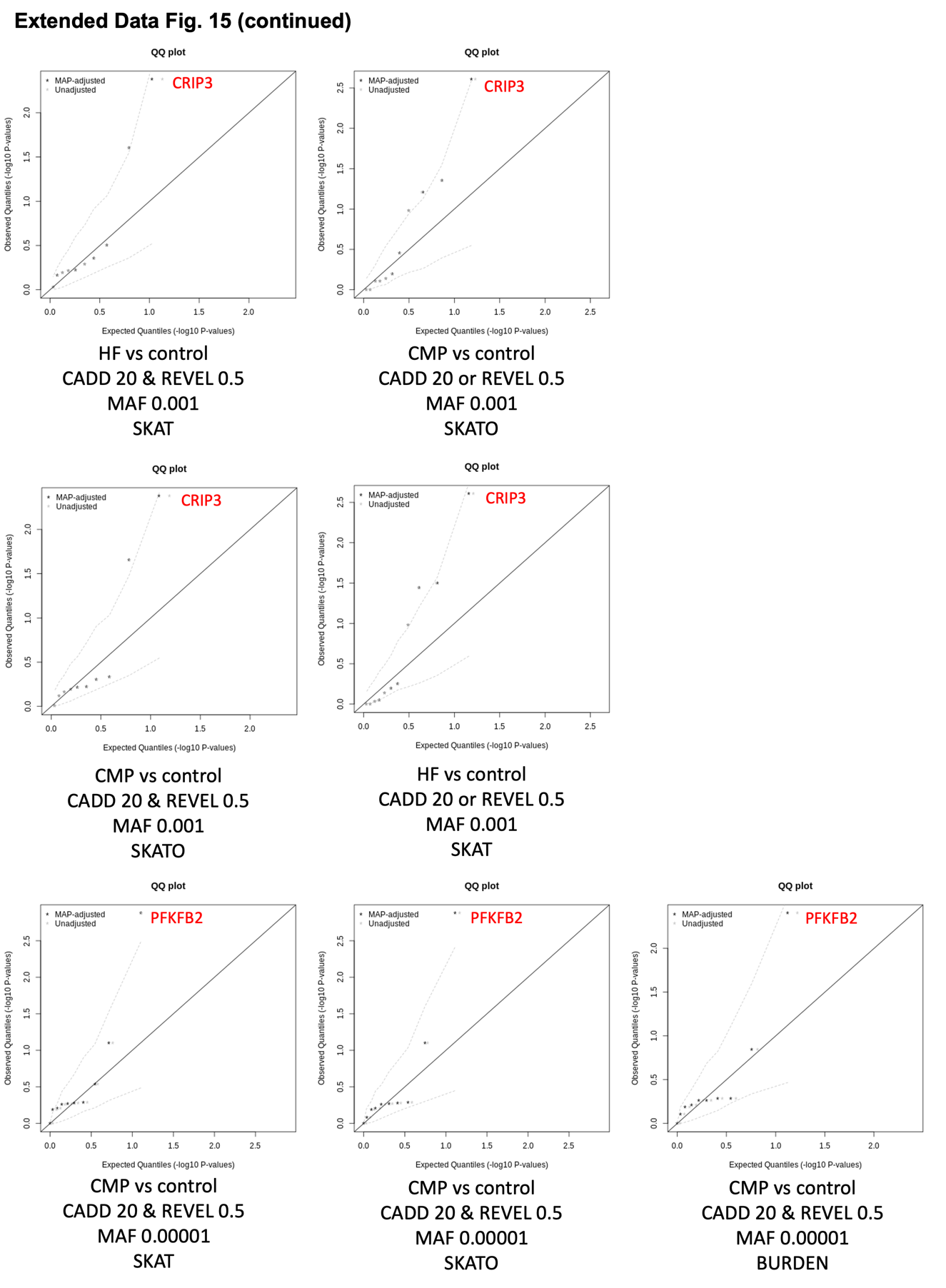


**Supplementary Fig.16.** **QQplots of rare-variant analysis comparing heart failure or cardiomyopathy cohort to control cohort from UK biobank** The number of cases was 1846, and the number of controls was 6857. HF, heart failure; CMP, cardiomyopathy; CADD, Combined Annotation Dependent Depletion; MAF, minimum allele frequency; SKAT, Sequence Kernel Association Test; SKAT-O, Optimal unified test. The number of cases was 1846 and that of controls was 6857.

**Supplementary Fig.17**

***Supplementary Fig.17 Bar plot showing the number of differentially expressed genes (DEGs) identified from the comparison of the presence or absence of treatments or comorbidities.*** *The right side of the plot represents the presence of the specified treatment or condition, and for gender, it represents male. Control_His, tissue with normal histology from cardiomyopathy as grouped in Figure 5; Diseased_ES, tissue with abnormal histology in end-stage heart failure as grouped in Figure 5; LVAD, Left ventricular assist device; RAAS inhibitor, Renin-angiotensin-aldosterone system inhibitor; ARNI, Angiotensin receptor-neprilysin Inhibitor; Hb, Hemoglobin; VT/VF, Ventricular tachycardia or ventricular fibrillation; RRT, Renal replacement therapy including hemodialysis and continuous renal replacement therapy.*

**Supplementary Fig.18**

**Supplementary Fig.18 Correlation heatmaps illustrating the impact of including treatments and comorbidities as covariates in differential gene expression analysis.** Each heatmap represents the correlation of logFC values across groups defined by clinical and histological features in cardiomyocytes (as shown in Figure 5), with treatments and comorbidities included as covariates (x-axis) and without these clinical factors as covariates (y-axis). The heatmaps display all genes analyzed, highlighting the overall correlation in logFC. Labels for specific treatments and comorbidities are positioned above their respective heatmaps for clarity. LVAD, Left ventricular assist device; RAAS inhibitor, Renin-angiotensin-aldosterone system inhibitor; ARNI, Angiotensin receptor-neprilysin Inhibitor; Hb, Hemoglobin; VT/VF, Ventricular tachycardia or ventricular fibrillation; RRT, Renal replacement therapy including hemodialysis and continuous renal replacement therapy.
