# Supplementary material for: Spatial transcriptional landscape of human heart failure
Source: Eur Heart J. 2025 May 8;46(31):3098–114. doi: 10.1093/eurheartj/ehaf272 (PMC12349961; doi:10.1093/eurheartj/ehaf272)
Supplement: ehaf272_Supplementary_Data [file ehaf272_supplementary_data.zip › SupplementaryMethods_EHJ2ndrevision_20250210.docx]

**Supplementary Methods**

**STUDY PARTICIPANT DETAILS**

The study provided detailed information about the participants for spatial transcriptomics in Table1 and Supplementary Table 1-2. Approval for this study was obtained from the Institutional Review Board (IRB) of Asan Medical Center in Seoul, Korea, under the protocol numbers 2020-0613 and 2020-1054, as well as from the IRB of Sejong General Hospital in Incheon, Korea, under the protocol number 2020-06-014-008. Informed consent was obtained from participants who were prospectively enrolled, ensuring that they were fully informed about the study's purpose, procedures, potential risks, and benefits before providing their consent. In cases where tissues and data were collected retrospectively, the IRB waived the requirement for informed consent, given that the data had been de-identified to protect participant privacy.

**METHOD DETAILS**

***Human tissue samples***

Adult human heart samples were harvested from a total of 44 subjects between January 2018 and April 2021 and kept in paraffin blocks after formalin fixation. Control samples were obtained from autopsy cases with no history of HF or extracted from the left ventricle of the patients with normal left ventricular function while receiving a heart transplant or a heart-lung transplant for isolated right ventricular failure or other reasons. Failing hearts were obtained from explanted hearts of recipients receiving a heart transplant (n=21), from the excisional biopsy of patients receiving a left ventricular assistant device (n=16), from the myectomy for obstructive HCM (n=9), and from autopsy (n=1). Samples from surgical procedures were from Asan Medical Center and Mediplex Sejong Hospital, and autopsy cases were purchased from Proteogenex. A pathologist reviewed H&E-stained slides from multiple regions of the left and right ventricles, and a total of 90 cores of 2 mm diameter were collected from 51 heart species. The pathologist first tried to select regions with the most representative histology for the clinical diagnosis and then searched for control regions in terms of histologic features in the corresponding ventricle when possible. Seventy-four cores were from the left ventricles, and 16 cores were from the right ventricles. We distributed them into two tissue microarray (TMA) blocks. Clinical information of age, sex, time of harvest, clinical diagnosis of HF, cause of death in case of the autopsy, co-morbidities, surgical procedure, left ventricular and right ventricular function measured by echocardiography, serum natriuretic peptide levels, and the time intervals from harvest to fixation were collected. The time intervals from harvest to fixation were classified into two groups: short (less than 4 hours) and extended (4-12 hours) for further analysis. Histologic features for each region of interest (ROI) were meticulously documented by a cardiac pathologist who was blinded to the corresponding RNA expression data. The pathologist provided a comprehensive initial description of histological characteristics, including normal appearance, degenerative changes, hypertrophy, fibrosis, myocyte disarray, among others. Following this extensive evaluation, a grading system was applied where hypertrophy, degeneration, and fibrosis were scored on a scale from 0 to 3, and myocyte disarray from 0 to 2. ROIs were then classified as “normal” if they exhibited no abnormal histologic findings, and as “abnormal” if any abnormality was present in order to perform combined analysis with clinical features. The relevant institutional review boards approved research using tissues at Asan Medical Center and Sejong General Hospital.

***GeoMx Digital Spatial Profiler experiment***

The slide sections from the TMA blocks went through the Nanostring GeoMx human Whole Transcriptome Atlas (WTA, NanoString Technologies, Seattle, WA) experiment^1^. Briefly, formalin-fixed, paraffin-embedded sections were deparaffinized, went through antigen retrieval procedures, and then were incubated with human WTA UV-cleavable RNA probes. They were co-stained with fluorescently conjugated antibody morphologic markers against Troponin I, Vimentin, and CD31 and were scanned with the Digital Spatial Profiler (DSP) instrument (NanoString Technologies, Seattle, WA) to produce digital fluorescent images of the falls. The pathologist (H.S.H) reviewed both immunofluorescent images and the H&E staining of the consecutive slides and selected the ROI. The combination of the antibodies identified the cell-type specific segmentations or area of interest (AOI): segmentation of cardiomyocytes was Troponin I positive, segmentation of endothelial cells was Vimentin and CD31 positive and Troponin I negative, segmentation of fibroblast was identified by Vimentin-positive, CD31 and Troponin I negative. Nuclei were counter-stained with DAPI. The pathologist finally selected 161 cell-type specific segmentations and 17 non-segmented ROIs from 90 cores. ROIs were segmented based on the expression of cell-type markers using the DSP auto-segmentation tool with manually tuned settings. Area of interest (AOI) stands for each cell-type specific segmentation in ROI, and because of the limitation in maximum numbers of AOIs in each slide and minimum required cell counts, we selected 98 cardiomyocyte AOIs, 49 endothelial cell AOIs and 14 fibroblast AOIs (Table2). We took 17 additional adjacent non-segmented ROIs with the same histologic features as the original corresponding ROIs in order to compare RNA expression profiles between cell-type specific segmentations and non-segmented ROIs since the GeoMx method does not provide non-segmented gene expression profiles from the segmented ROIs.

***Quality control, batch correction and normalization***

Count data were processed using the NanoString DSP software. The representative thresholds for segment quality control (QC) were raw read threshold 1000, aligned read threshold 50, sequencing saturation threshold 50, surface area threshold 5000, and nuclei count threshold 30. No segments were filtered out through this segment QC. The representative QC thresholds for probes were the ratio of probe geomean across all segments 0.1, the percent of AOIs threshold for Grubbs test 20, and the standard deviation amount for the LOQ 2. Since the batch effect introduced by separate experiment for each slide was detected with BatchQC R package v1.16.3 (<https://bioconductor.org/packages/release/bioc/html/BatchQC.html>), it was corrected using ComBat-seq in sva R package v3.36.0^2^ (https://bioconductor.org/packages/release/bioc/html/sva.html) (Supplementary Fig.3). Counts were normalized by Q3 normalization, and outlier AOIs were filtered out based on the PCA plot in each comparison.

***Immunohistochemical staining and scoring***

Slides from TMA blocks, which were used in our GeoMx experiment, were also stained with primary antibodies in *KEY RESOURCE TABLE*. Immunohistochemistry was performed by Woodang Network Co.,Ltd (Gangwon-do, Korea). The results were interpreted by pathologists (G.Y.L., and H.S.H.) blinded to ROI information. The endothelial staining of PLVAP, ACKR1 or CCL14 visible at 40x magnification was scored as 2 in terms of staining strength while their staining visible at higher magnification was scored as 1. If the staining was visible in more than 25% of the core stained, it was scored as 2 in terms of extent and if it was visible in less than 25% of the core, it was scored as 1. If no staining of endothelial cells were observed, both strength and extent score were 0. For other markers or myocardial staining of CCL14 was assessed with Histoscore (H-score). H-score was calculated as following formula: H-score = sum (Pi × i), where Pi represents the percentage of cells stained at intensity i (ranging 0-100) and i represents the staining intensity (ranging from 0 to 3).

Multiplex immunohistochemistry was performed by prismCDX Co., Ltd (Gyeonggi-do, Korea) to visualize the co-localization of ACKR1, PLVAP, and the endothelial cell marker CD34, and to measure the density of these cells in relation to fibrosis. The staining was conducted on slides from TMA blocks, which were also used in our GeoMx experiment. Additionally, multiplex immunohistochemistry was carried out to assess the co-localization of CD31, Vimentin, CD45, and alpha-SMA in perivascular area in heart tissue on slides from a tissue sample from the subject labeled AMC_008. Details of the antibodies used are provided in the KEY RESOURCE TABLE. The samples were prepared using 4-μm FFPE sections. After heating at 60℃, slides underwent sequential multiplex immunofluorescence staining on a Leica Bond Rx™ stainer. Antigen visualization was achieved with tyramide signal amplification, followed by DAPI for nuclei staining. Slides were imaged at 20x on the PhenoImager^TM^ HT (Akoya Biosciences). A representative image for training were selected in Phenochart (Akoya Biosciences), and an algorithm was created in the inForm Image Analysis software (Akoya Biosciences). Multispectral images were unmixed using the spectral library in inFome software. Based on DAPI staining, each single cell was segmented, and phenotyping was performed according to the expression compartment and intensity of each marker. After designating the region (ROI, region of interest) to be analyzed on the tissue slide, the same algorithm created in this way was applied and batch-running. The exported data is consolidated and analyzed in R studio (4.1.2 version) using the phenoptr (Akoya Biosciences) and phenoptrReport (Akoya Biosciences) packages.

***Rare variant analysis on UK biobank whole exome sequencing cohort.***

Rare variant analysis was performed using UK biobank final exomes released in July 2022. HF and control cohorts were defined by ICD9, ICD10, OPCS3, OPCS4 and UK biobank Field 6150, 20002, 20004, and 42000 as described in Supplementary Table 20. Those diagnosed with DCM, HCM, ICM and other HF before an age of 60 without secondary causes such as valvular heart disease, myocarditis, congenital heart disease, hypertension, or renal failure were defined as the HF cohort. Those died after an age of 70 without significant cardiac problems related to HF were considered as the control cohort.

Among the 23 genes significantly up-regulated in Control_His compared to Control_Clin (logFC>0.5 and adjusted *p-*value<0.05), we test 12 genes down-regulated in Diseased_ES compared to Control_His (logFC<0 and *p-*value<0.05). Rare variant analysis was performed using R package SKAT 2.2.5.

***Cell decomposition and validation analysis using external snRNA-seq transcriptomic profile***

To validate our marker selection rationale, gene expression profiles, and the characterization of ACKR1 and PLVAP double-positive endothelial cells, we utilized the sc/snRNA-seq data from Koenig et al ^3^. This dataset was also employed to generate the cell profile matrix for cell deconvolution analysis. We specifically chose single nucleus data to mitigate issues of cellular heterogeneity for the analysis involving cardiomyocytes. For endothelial cell subclustering analysis sc/snRNA-seq data were utilized. The dataset includes samples from 18 DCM patients and 27 control subjects for all data, with 13 DCM patients and 25 control samples specifically for single nucleus data. The dataset was downloaded from the NIH Gene Expression Omnibus (GEO) repository (<https://www.ncbi.nlm.nih.gov/geo/query/acc.cgi?acc=GSE183852>). We performed downstream analysis using the existing Seurat object, which had already pre-processed quality control and normalization. Detailed downstream analysis methods are described below.

**QUANTIFICATION AND STATISTICAL ANALYSIS**

All statistical data analyses and visualizations were conducted with R4.0.0.

***Principal component analysis***

Principal component (PC) analysis was performed using PCAtools 2.6.0 (<https://www.bioconductor.org/packages/release/bioc/html/PCAtools.html>). Calculated PC1, PC2, and PC3 were used to create 3D PCA plots with R package plotly 4.10.0. The correlation between PCs and clinical variables was done with the eigencorplot function in R.

***Heatmap generation***

Heatmaps were generated using the ‘Heatmap’ function in the R package ComplexHeatmap_2.10.0. (<https://www.bioconductor.org/packages/release/bioc/html/ComplexHeatmap.html>). For the heatmaps, Z-scores were calculated using the formula (value−mean)/standard deviation. This approach was applied to curated marker genes from the literature (Figure 2f) and the top 4 genes with the highest log fold change (logFC) from the DEGs comparing each segment to the other segments (Figure 2g). For heatmaps comparing clinical phenotypes to controls in both endothelial cells and cardiomyocytes, scaled log counts per million (cpm) were used for the top and bottom 20 genes from the DEG analysis. Additionally, heatmaps were generated based on DEGs across cell types to demonstrate the clustering of segments according to cell types. In this case, scaled log counts per million (cpm) were used for the top and bottom 100 genes from the DEGs comparing each segment to the other segments.

***Differential expression analysis***

Differential expression testing was performed by cell types, clinical diagnosis of cardiomyopathy, histological features, and groups classified by both clinical and histological information. After QC, batch correction, and normalization, we applied a linear mixed effect model using the DREAM method in variancePartition R package v1.25.13 (<http://bioconductor.org/packages/release/bioc/html/variancePartition.html>)^4^. Patient id, institutes where the samples originated, and the time interval from harvest to fixation were included as random effect variables when appropriate. The Kenward-Roger method for the low sample size was used. Two-sided *p*-values were calculated. A Benjamini–Hochberg correction was applied for multiple testing corrections, and an FDR-adjusted *p*-value < 0.05 was used for significance.

***Gene set analysis***

We performed gene set enrichment analysis (GSEA) using R package fgsea 1.14.0, clusterProfiler 4.2.1, and enrichplot 1.14.1. To elucidate the gene sets related to the PC1 of the whole cohort, we ranked genes according to PC1 loading and performed GSEA for Gene Ontology^5^ with the classic method (unweighted). We performed an GSEA of Hallmark pathways^6^ by clinical diagnosis, histological features, and groups classified by clinical and histological features. We calculated scores by multiplying the direction (sign) of fold change and minus the logarithm of the *p*-value for each gene from the differential expression test (sign(logFC) * -log10(p-value) in R code), and genes were ranked and weighted with the scores (p=1) (<https://baderlab.github.io/Cytoscape_workflows/EnrichmentMapPipeline/supplemental_protocol1_rnaseq.html>)^7^.

***Spatial Deconvolution***

Spatial deconvolution was performed using the R package SpatialDecon 1.4.3^8^. The cell profile matrix was generated using the same snRNA-seq data from Koenig et al. employed in the replication analysis described above ^3^. Marker genes associated with the different cell types identified in this study were determined using the 'FindAllMarkers' function in the R package Seurat. Endocardium and epicardium cell markers were excluded since these cell types were morphologically excluded during the selection of ROIs.

To enhance the specificity of each cell type profile, only genes with a |logFC| > 1 and an adjusted p-value < 0.05 were selected. Normalization was performed using the counts data of the corresponding genes in the snRNA-seq dataset, facilitated by the 'create_profile_matrix' function in SpatialDecon, which was then used to generate the cell profile matrix.

Using this cell profile matrix as markers and the normalized gene expression data from the GeoMx DSP as the input expression matrix, the 'spatialdecon' function in SpatialDecon was applied to calculate the proportions of different cell types within each AOI.

***Validating gene expression profiles using external snRNA-seq transcriptomic profile***

To validate our AOI gene profiles, we correlated them with external snRNA-seq data from Koenig et al ^3^. Highly expressed genes in each cluster were identified using the 'FindAllMarkers' function in Seurat, applying the Wilcoxon signed-rank test. We focused on genes with |logFC| > 1 and an adjusted p-value < 0.05, and among these, only the genes included in our GeoMx analysis were selected for further analysis. Average expression for each cluster in the snRNA-seq data was calculated using the 'AverageExpression' function in Seurat. We then determined the correlation between these average expressions and the log-normalized counts of the same genes in our data using the R package corrplot 0.92.

***Uniform manifold approximation and projection* (*UMAP) analysis for marker gene expression in specific cell types***

To visualize cell populations and AOIs within heart tissue, we performed UMAP analysis on our data and snRNA-seq data from Koenig et al ^3^. For the GeoMx dataset, we utilized the R package umap 0.2.8.0 (https://github.com/tkonopka/umap?tab=readme-ov-file) to generate UMAP plots. For the snRNA-seq data, nuclei were clustered based on their gene expression profiles using the Seurat package in R, identifying distinct populations such as cardiomyocytes, endothelial cells, fibroblasts, and others, each represented by different colors. Feature plots were generated to display the expression levels of key marker genes, including TNNI3, PECAM1, and VIM, using a color gradient to highlight their distribution within the clusters.

***Emulating segmentation strategy and its effect on PCA plot using snRNA-seq***

To ensure the robustness of our segmentation strategy, we emulated it using snRNA-seq data. We performed pseudo-bulk PCA at the patient level, aggregating single cells based on the normalized average expression levels of marker genes to mirror our antibody-based cell selection strategy. For the Cardiomyocyte AOI in our dataset, cells with normalized TNNI3 expression > 0.5 and normalized VIM and CD31 expression < 0.5 were aggregated for each individual. To replicate the fibroblast AOI, cells with normalized TNNI3 and CD31 expression < 0.5 and normalized VIM expression > 0.5 were aggregated. For endothelial cells, we compared two strategies: using normalized CD31 expression > 0.5 alone or normalized CD31 expression > 0.5 combined with normalized VIM expression > 0.5, reflecting our original approach. We created subsets of these three subclustered cell populations and generated new Seurat objects for each emulated AOI using the ‘AverageExpression’ function in Seurat for each sample. The R package ggfortify 0.4.17 was utilized to compute principal components and generate the PCA plot.

***Endothelial subclustering and ACKR1 and PLVAP double positive endothelial cell population***

We performed subclustering exclusively on the "Endothelium." Each sample was normalized individually using the SCTransform 0.3.5 package in R, and then all samples were merged into a combined dataset. To mitigate batch effects, we utilized the Harmony 0.1.1 package. We selected 3,000 highly variable genes to align cells from diverse sample origins. PCA was performed for cell clustering, and the results were visualized using UMAP with the top 40 principal components.

To identify differentially expressed genes (DEGs), we used the 'FindAllMarkers' function in Seurat, applying the Wilcoxon signed-rank test, and annotated each cluster using known markers. For cluster proportion comparisons, statistical tests were performed using the Mann-Whitney U test. To assess the characteristics of the 'Endothelial cell inflammation' cluster, we selected DEGs with logFC > 0.25 and an adjusted p-value < 0.05. Subsequently, we applied EnrichR for gene set enrichment analysis using Hallmark gene sets.

***Cell-cell interaction analysis***

We used CellPhoneDB as the database for ligand-receptor interactions^9^.The analysis was conducted by dividing samples based on the degree of histological differences in degeneration, fibrosis, hypertrophy, and disarray, then calculating interactions between segments, excluding the non-segmented ROIs. The "significant_means" output file from CellPhoneDB was manually curated to systematically organize pairs such that gene 1 represents ligand encoding and gene 2 represents receptor encoding. Interactions with a p-value below 0.05 were considered significant, as recommended by the default CellPhoneDB algorithm. Interaction pairs showing significance when histologically progressing from grade 0,1 to grade 2,3 (grade 0 to grade 1,2 for disarray) were sorted and visualized using the R package ktplots 2.3.0. The dot size and color represent the scaled means, with significant cases outlined in a black border.

***Correlation in the variation of gene expression explained by clinical diagnosis and histological features.***

We selected 500 genes with high variation in gene expression and calculated the proportion of variation explained by the clinical diagnosis and histological features using variancePartition R package 1.25.13. The correlation between the variables was measured with Pearson’s coefficients and illustrated with R package corrplot 0.92.

**Data Availability**

The data associated with this study has been submitted to the GEO repository under the accession number GSE271676 and will be publicly available upon official publication of the paper. Additionally, the data and accompanying code can be accessed through the following link: [Mendeley Data](https://data.mendeley.com/datasets/gmgcdhmmdx/draft?a=a30a4227-b205-471d-b16f-312d5edfbb8e). Further information and requests for resources can be directed to the lead contact, Sang Eun Lee, at [sangeunlee.md@amc.seoul.kr](mailto:sangeunlee.md@amc.seoul.kr) or [sangeunlee.md@gmail.com](mailto:sangeunlee.md@gmail.com).

**KEY RESOURCES TABLE**

| REAGENT or RESOURCE | SOURCE | IDENTIFIER |
| --- | --- | --- |
| Spatial transcriptome raw data | *https://data.mendeley.com/datasets/gmgcdhmmdx/draft?a=a30a4227-b205-471d-b16f-312d5edfbb8e* |  |
| Staining raw data | *https://data.mendeley.com/datasets/gmgcdhmmdx/draft?a=a30a4227-b205-471d-b16f-312d5edfbb8e* |  |
| All original code | *https://data.mendeley.com/datasets/gmgcdhmmdx/draft?a=a30a4227-b205-471d-b16f-312d5edfbb8e* |  |
| Materials for staining (antibodies, etc) |  |  |
| Troponin I | Abcam | AB196384 |
| PECAM-1/CD31(for GeoMx) | Abcam | AB215912 |
| Vimentin | Santa Cruz | sc-373717 |
| ACKR1​ | Sigma | HPA017672 |
| PLVAP​ | Novus | NBP1-83911 |
| CCL14​ | Santacruz | sc-28388 |
| SLC6A6​ | Invitrogen | PA5-53431 |
| RRAD​ | Sigma | HPA041755 |
| IGFBP2​ | Sigma | HPA035145 |
| UCHL1​ | Sigma | HPA005993 |
| MYOM1​ | Sigma | HPA014305 |
| CD34 | Abcam | AB81289 |
| DAPI | Thermo Scientific | 62248 |
| CD45 | Cell Signaling Technology | 13917 |
| alpha-SMA | Abcam | ab5694 |
| CD31 (for Multiplex IHC) | Abcam | ab28364 |
| Vimentin (for Multiplex IHC) | Cell Signaling Technology | 5741 |
| BOND Polymer Refine Detection | Leica biosystems | DS9800 |

**References**

1. Zimmerman SM, Fropf R, Kulasekara BR, Griswold M, Appelbe O, Bahrami A*, et al.* Spatially resolved whole transcriptome profiling in human and mouse tissue using Digital Spatial Profiling. *Genome Research* 2022;**32**:1892-1905. doi: 10.1101/GR.276206.121/-/DC1

2. Zhang Y, Parmigiani G, Johnson WE. ComBat-seq: batch effect adjustment for RNA-seq count data. *NAR Genomics and Bioinformatics* 2020;**2**. doi: 10.1093/NARGAB/LQAA078

3. Koenig AL, Shchukina I, Amrute J, Andhey PS, Zaitsev K, Lai L*, et al.* Single-cell transcriptomics reveals cell-type-specific diversification in human heart failure. *Nature Cardiovascular Research* 2022;**1**:263-280. doi: 10.1038/s44161-022-00028-6

4. Hoffman GE, Roussos P. Dream: powerful differential expression analysis for repeated measures designs. *Bioinformatics* 2021;**37**:192-201. doi: 10.1093/BIOINFORMATICS/BTAA687

5. Ashburner M, Ball CA, Blake JA, Botstein D, Butler H, Cherry JM*, et al.* Gene Ontology: tool for the unification of biology. *Nature genetics* 2000;**25**:25-25. doi: 10.1038/75556

6. Liberzon A, Birger C, Thorvaldsdóttir H, Ghandi M, Mesirov JP, Tamayo P. The Molecular Signatures Database (MSigDB) hallmark gene set collection. *Cell systems* 2015;**1**:417-417. doi: 10.1016/J.CELS.2015.12.004

7. Reimand J, Isserlin R, Voisin V, Kucera M, Tannus-Lopes C, Rostamianfar A*, et al.* Pathway enrichment analysis and visualization of omics data using g:Profiler, GSEA, Cytoscape and EnrichmentMap. *Nat Protoc* 2019;**14**:482-517. doi: 10.1038/s41596-018-0103-9

8. Danaher P, Kim Y, Nelson B, Griswold M, Yang Z, Piazza E*, et al.* Advances in mixed cell deconvolution enable quantification of cell types in spatial transcriptomic data. *Nature Communications* 2022;**13**. doi: 10.1038/s41467-022-28020-5

9. Efremova M, Vento-Tormo M, Teichmann SA, Vento-Tormo R. CellPhoneDB: inferring cell-cell communication from combined expression of multi-subunit ligand-receptor complexes. *Nat Protoc* 2020;**15**:1484-1506. doi: 10.1038/s41596-020-0292-x
