# Supplementary material for: Spatial transcriptional landscape of human heart failure
Source: Eur Heart J. 2025 May 8;46(31):3098–114. doi: 10.1093/eurheartj/ehaf272 (PMC12349961; doi:10.1093/eurheartj/ehaf272)
Supplement: ehaf272_Supplementary_Data [file ehaf272_supplementary_data.zip › SupplementaryTableList_EHJ2ndrevision_20250210.docx]

**Supplementary Tables**

Supplementary Table 1. Treatments, comorbidities and laboratory findings of patients at each instance of tissue collection

Supplementary Table 2. Procedures for tissue collection across clinical phenotypes

Supplementary Table 3. Component loadings of PC1-5

Supplementary Table 4. Differentially expressed genes across cell types

Supplementary Table 5. Differentially expressed genes in cardiomyocytes according to clinical diagnosis

Supplementary Table 6. Differentially expressed genes in endothelial cells according to clinical diagnosis

Supplementary Table 7. Treatments, comorbidities and laboratory findings of cardiomyocyte segments according to clinical diagnosis

Supplementary Table 8. Treatments, comorbidities and laboratory findings of endothelial segments according to clinical diagnosis

Supplementary Table 9. Differentially expressed genes in cardiomyocytes and endothelial cells according to histology

Supplementary Table 10. Treatments, comorbidities and laboratory findings of cardiomyocyte segments according to histology

Supplementary Table 11. Treatments, comorbidities and laboratory findings of endothelial segments according to histology

Supplementary Table 12. Differentially expressed genes in cardiomyocytes according to combined clinical and histologic features

Supplementary Table 13. Treatments, comorbidities and laboratory findings of cardiomyocyte segments according to combined clinical and histologic features

Supplementary Table 14. Differentially expressed genes in endothelial cells according to combined clinical and histologic features

Supplementary Table 15. Treatments, comorbidities and laboratory findings of endothelial segments according to combined clinical and histologic features

Supplementary Table 16. Rare-variant analysis of the UK biobank cohort on genes up-regulated in the Control_His group compared to the Control_Clin group and down-regulated in the Diseased_ES group compared to the Control_His

Supplementary Table 17. Differentially expressed genes in cardiomyocytes from the Control_His or the Diseased_ES groups categorized by treatments and comorbidities.

Supplementary Table 18. Differentially expressed genes in endothelial cells from the Diseased_ES group categorized by treatments and comorbidities.

Supplementary Table 19. Detailed sample numbers used in differential expression analysis in Figure 3 and Figure 4.

Supplementary Table 20. Terminology and criteria to define heart failure, cardiomyopathy and control cohort in UK biobank
